# FEM Numerical Calculations and Experimental Verification of Extrusion Welding Process of 7075 Aluminium Alloy Tubes

**DOI:** 10.3390/ma19010075

**Published:** 2025-12-24

**Authors:** Dariusz Leśniak, Konrad Błażej Laber, Jacek Madura

**Affiliations:** 1 Department of Materials Science and Non-Ferrous Metals Engineering, Faculty of Non-Ferrous Metals, AGH University of Science and Technology, Adama Mickiewicza 30 Ave., 30-059 Krakow, Poland; madura@agh.edu.pl; 2 Department of Metallurgy and Metal Technology, Faculty of Production Engineering and Materials Technology, Czestochowa University of Technology, 19 Armii Krajowej Ave., 42-200 Czestochowa, Poland; konrad.laber@pcz.pl

**Keywords:** extrusion, aluminium alloys, FEM numerical modelling, material models, extrusion force, product quality

## Abstract

Extrusion of AlZnMgCu alloys is associated with a very high plastic resistance of the materials at forming temperatures and significant friction resistance, particularly at the contact surface between the ingots and the container. In technological practice, this translates into high maximum extrusion forces, often close to the capacity of hydraulic presses, and the occurrence of surface cracking of extruded profiles, resulting in a reduction in metal exit speed (production process efficiency). The accuracy of mathematical material models describing changes in the plastic stress of a material as a function of deformation, depending on the forming temperature and deformation speed, plays a very important role in the numerical modelling of extrusion processes using the finite element method (FEM). Therefore, three mathematical material models of the tested aluminium alloy were analysed in this study. In order to use the results of plastometric tests determined on the Gleeble device, they were approximated with varying degrees of accuracy using the Hnsel–Spittel equation and then implemented into the material database of the QForm-Extrusion^®^ programme. A series of numerical FEM calculations were performed for the extrusion of Ø50 × 3 mm tubes made of 7075 aluminium alloy using chamber dies for two different billet heating temperatures, 480 °C and 510 °C, and for three different material models. The metal flow was analysed in terms of geometric stability and dimensional deviations in the wall thickness of the extruded tube and its surface quality, as well as the maximum force in the extrusion process. Experimental studies of the industrial extrusion process of the tubes, using a press with a maximum force of 28 MN and a container diameter of 7 inches, confirmed the significant impact of the accuracy of the material model used on the results of the FEM numerical calculations. It was found that the developed material model of aluminium alloy 7075 number 1 allows for the most accurate representation of the actual conditions of deformation and quality of extruded tubes. Moreover, the material data obtained on the Gleeble simulator made it possible to determine the limit temperature of the extruded alloy, above which the material loses its cohesion and cracks appear on the surface of the extruded profiles.

## 1. Introduction

Extrusion of difficult-to-deform AlZnMg(Cu) aluminium alloys is extremely difficult in industrial practice because of the high yield strength of the material under extrusion conditions and its high friction resistance, which leads to high extrusion forces and high stresses acting on the die (strong elastic deflection of the die) and high unevenness of metal flow. As a result, high geometric instability of the extruded product is observed, with dimensional deviations exceeding the permissible limits, and in extreme cases, loss of material cohesion and numerous surface cracks in the product.

The available scientific literature contains many articles on issues related to force in the aluminium alloy extrusion process and product quality associated with the uniformity of flow and the temperature of the extruded metal. Most often, optimisation concerns the design of the die in terms of minimising the extrusion force or controlling the speed of aluminium flow through the die. The vast majority of these works concern easily deformable aluminium alloys, aluminium composite materials, or magnesium alloys.

The results of research on the analysis of force in the extrusion process of aluminium alloys can be found in [1,2]. In order to ensure the feasibility of the process and the quality of the finished product, it is expected that the extrusion force must be as low as possible [1]. The authors of that paper performed calculations to determine the extrusion force of 6xxx series aluminium alloys with a view to minimising it for conical flat dies. In the actual extrusion process, it often happens that the force is too high, which poses a serious threat to production. It increases the unstable factors in metal forming and the extrusion difficulties, and moreover, it can easily cause premature damage to tools, in particular the die, and failure of the extrusion equipment system [2]. The authors of that paper attempted to reduce the extrusion force of AlMgSi alloys using an original porthole die design. Their research focused mainly on materials and techniques. The die construction, which can reduce the extrusion force, was analysed in detail and can be used as a reference point in die design.

In work [3], numerical modelling was used to determine, among other things, the maximum force during the extrusion of thin rods from a biocompatible magnesium alloy containing a calcium additive. In turn, in [4], the authors performed an analytical and numerical analysis of, among other things, the extrusion force and stresses in the extrusion process of Al6063-SiC composite. Article [5] presents the detailed results of a numerical analysis of the hot extrusion process of twist drills, with careful consideration of thermal phenomena. As a result of that research, it was possible to determine the force parameters, the distributions of effective deformation, reduced stress, and temperature. As a result, it was proven that it is possible to produce twist drills by using extrusion. In [6], a detailed numerical analysis of stresses, forces, and temperatures arising during the axisymmetric cold extrusion of perforated aluminium alloy samples was carried out. In addition, the influence of the friction coefficient on effective stresses in samples with holes of various sizes located on the axis of symmetry was analysed. A comparative analysis of the extrusion forces acting on the punch at different friction coefficients was performed. The temperatures obtained during the extrusion of perforated samples without friction and in the presence of friction were compared. For a thorough analysis of the issue, the results of the extrusion of perforated samples were compared with the results obtained in samples without holes.

Article [7] presents the results of a numerical analysis of deformation force calculations performed on backward extrusion with the use of active friction force action. It was shown that the numerical methods of the analysis based on the finite element method gave accurate results. This allows us to recommend numerical methods for the calculation of the force values acting on a working tool in backward extrusion. In addition, numerical research enables the prediction of shape change in workpieces at any stage of the backward extrusion process. The force changes, calculated analytically and numerically, agree well with the experimental data. Then, in work [8], a new method of backward extrusion using small diameter billet is proposed. The die setup consists of three main parts: the fix-punch, the movable punch, and the die. The backward extrusion process was analysed using experimental and finite element (FE) methods. Based on the results, it was found that the load was reduced to less than a quarter of that seen in the conventional backward extrusion process. This is a result of reducing the cross-section of the initial billet. It was shown that the applied plastic strain in this new process is about two times higher than that in the conventional backward extrusion process.

In work [9], using the example of extrusion of a round bar, the results of a comparative analysis of the effect of the extrusion ratio on, among other things, the extrusion force are presented. The tests were carried out over a wide range of extrusion ratios with a constant external diameter of the die. Based on the results obtained, it was found, among other things, that as the extrusion ratio increases, the extrusion force increases; however, the increase in this force is not proportional to the increase in the extrusion ratio (extrusion force can be described by a logarithmic equation). The most intense increase in force was observed in the range of small extrusion ratios (max. 3).

An equally important technological parameter that directly affects the extrusion force is the temperature of the extruded material. Accurate determination and verification of temperature values in extrusion processes using numerical modelling methods is a complex issue. This is due, among other things, to the changing friction conditions during the extrusion process. The temperature value and distribution during extrusion are influenced by many factors, such as the cross-sectional area and length of the billet, degree of reduction and cross-sectional shape of the extruded product, extrusion speed, and extrusion method (technique). The temperature of the extruded material (and cooling conditions) also directly affects the microstructure and properties of the finished product.

The analysis of temperature in extrusion processes was addressed, among others, by the authors of works [10,11,12,13,14]. In work [10], the influence of temperature and extrusion ratio on the microstructure and mechanical properties of a new Al-4Cu-1Li-0.4Mg-0.4Ag-0.11Zr alloy was investigated. It was concluded that the softening mechanism during extrusion was dominated mainly by dynamic recovery (DRV). However, the microstructure showed significant dynamic recrystallisation (DRX) properties when the extrusion temperature increased to 480 °C. The texture orientation of the extruded rods was concentrated in the <111> and <001> directions, with the <111> texture showing greater intensity. It was stated that changes in the extrusion ratio and temperature have little effect on the type of texture but significantly alter its intensity. After the T6 ageing treatment, the density of the T1 phase increased with increasing extrusion ratios, accompanied by a finer size and more uniform distribution. High temperatures led to visible recrystallisation and grain coarsening, reducing deformability and plasticity. By optimising the temperature and extrusion ratio, a good combination of strength and ductility was ultimately achieved for the alloy under investigation. In turn, paper [11] presents results on the influence of temperature and die angle on the tensile strength, hardness, extrusion load, and yield stress of aluminium 6063 processed by the Equal Channel Angular Extrusion Method (ECAE). Based on the results obtained, it was found, among other things, that for all analysed temperatures and angles, temperature has a greater influence on the ECAE process parameters than die angle. High temperatures can also reduce the force required for extrusion. A high temperature of the extruded material also allows for a more uniform distribution of yield stresses in aluminium processed by the ECAE.

As emphasised by the authors of the study [12], extrusion temperature and speed are important factors influencing microstructure development. In turn, microstructure development plays a key role in maintaining the mechanical properties of materials. In direct extrusion, the homogeneous evolution of the microstructure along the length of the extruded bar may be disrupted due to the non-isothermal evolution of the initial temperature. Therefore, a new solution with real-time control of extrusion temperature and speed was proposed to characterise the effect of temperature on the microstructure and to obtain its uniform development for magnesium alloy. During extrusion, the temperature of the bar was monitored using a thermal imaging camera. The extrusion speed was controlled in real time, depending on the temperature difference between the set reference temperature and the temperature obtained from the thermal imaging camera. Based on the results obtained, it was found that real-time extrusion speed control effectively influenced the development of the microstructure at a stable temperature.

In work [13], the influence of temperature and pressure changes on deformation prediction using the finite element method (FEM) in the extrusion process was investigated. Based on the results obtained, it was found, among other things, that FEM can be successfully applied to model the deformation patterns in the load/displacement traces and temperature evolution during the extrusion cycle.

The authors of paper [14] also dealt with modelling the temperature distribution during the extrusion process using FEM. Experimental verification confirmed the high accuracy obtained by the computer simulations. The research and computer simulations carried out showed that the accurate selection of the rheological model and the determination of its parameters affect the accuracy of the results obtained.

In the case of plastic working processes, the key characteristic determining the plastic forming ability of a given material is the yield stress σ_p_ and the limit strain ε_l_ [15]. The yield stress under uniaxial stress conditions is a function of strain (ε), strain rate ($\dot{\varepsilon }$), temperature (T), and strain history. Determining the rheological properties of materials is particularly difficult under hot plastic working conditions, as the material structure undergoes simultaneous strengthening processes resulting from the plastic deformation mechanism, the presence of foreign phase particles in solution and in the form of precipitates, as well as thermally activated softening processes that lead to material weakening [15,16]. For the correct determination of the rheological properties of the analysed material, it is important to take into account the influence of material temperature, strain values, and strain rate, which increase the accuracy of calculations [17,18].

The accuracy of mathematical models describing changes in yield stress depending on deformation parameters is important in processes characterised by high deformation values, such as extrusion or twisting. This is confirmed by the results of published studies, including, among others [19,20]. These studies demonstrate the significant impact of the accuracy of the mathematical model of rheological properties on the distribution and values of the deformation parameters and on the stress of the tested material. The research clearly shows that the correct calculation of deformation parameters (deformation value, deformation rate) and stress requires the use of an accurate mathematical model of the tested material. In turn, accurate calculation of the yield stress value is necessary for the correct determination of the energy and force parameters of various plastic working processes, e.g., extrusion force, metal pressure force on the roll during rolling, force in the drawing process, etc. Furthermore, according to the authors of [19,20], the correct determination of deformation and stress parameters in a material, which depends to a large extent on the accuracy of the corresponding mathematical model, is particularly important during the numerical analysis of complex deformation states and in the case of tests whose parameters exceed the research capabilities of the equipment used.

Numerous results of plastometric tests aimed at determining rheological properties and developing material models for the numerical modelling of extrusion processes have been published, including, among others [21,22,23,24].

There are few works in the available technical literature that deal with predicting the extrusion force of difficult-to-deform 7075 aluminium alloys through porthole dies and, at the same time, the uniformity of metal flow and its impact on the quality of extruded profiles. One of the few is the work by Thanh-Cong Nguyen [25], which analysed the impact of porthole dies designed using FEM on the uniformity of metal flow from the die opening and the geometric stability of an extruded profile made of 7075 aluminium alloy with a rather complex cross-sectional shape. The stress conditions of the tools and the force in the extrusion process were also predicted.

Paper [26] presents a geometrically non-linear theory of shells, dedicated to the analysis of thin-walled tubular shells with both open and closed cross-sections, which are susceptible to significant geometric non-linearities. The aim is to accurately model their structural response under conditions of large displacements and rotations, assuming small deformations. The proposed formulation is geometrically rigorous, meaning that there are no approximations in terms of the magnitude of kinematic variables. A 3D model using internal geometry and a moving reference frame approach was used. A key advantage is that the theory transcends the traditional distinction between beam and shell models by incorporating cross-sectional deformations. This includes both in-plane and out-of-plane warping. To ensure practicality and physical clarity, the paper deliberately avoids index notation and complex Riemann curvature tensors. The model was validated on two case studies through numerical simulations and comparison with the Finite Element Method (FEM). The analysis included ovalization by bending a circular tube and a U-shaped tube experiencing severe in-plane warping. The results showed strong convergence with FEM, confirming the theory’s ability to accurately capture large cross-sectional deformations in both configurations.

The research conducted in paper [27] constitutes a formal formulation and verification of the extended Generalised Beam Theory (GBT) for the analysis of thin-walled bars with open, curved cross-sections. The kinematics were defined by decomposing the displacement field into the product of axial amplitudes and cross-section-dependent trial functions. Plane (distortion) modes describing the deformation of the cross-section were selected as dynamic modes of an auxiliary curved beam mapping the cross-section’s contour. Out-of-plane (buckling) modes were then derived from the imposition of an internal constraint, i.e., the non-deformability of the central surface of the walls under shear. The differential equations of equilibrium were formalised using Hamilton’s principle and the variational method. This allowed for the effective consideration of the coupled phenomena of warping and distortion. The model was applied and verified on the example of a beam with a semi-annular cross-section with constant curvature. A comparison of the analytical results of GBT with those obtained using the finite element method (FEM) showed high consistency, confirming the accuracy of the proposed formulation. The research provides an advanced theoretical model for the precise analysis of the stability and dynamics of this type of complex structure.

However, there is a lack of technical literature analysing the impact of the accuracy of mathematical material models on the accuracy of numerical modelling of technological processes. Therefore, in the authors’ opinion, the research topic addressed in this paper is relevant. One of the novelties of this work in relation to previously published works is the use of three material models of the tested 7075 aluminium alloy, developed with varying degrees of accuracy, to determine the most important parameters of the extrusion process and the quality of the product. The research confirmed the significant impact of the accuracy of the material model used on the results of numerical calculations. Ultimately, a high degree of consistency was achieved between the theoretical and experimental extrusion forces, as well as the uniformity of metal flow, with impacts on the dimensional accuracy of the extrudates.

## 2. Materials and Methods

### 2.1. Materials

The tests presented in the paper were performed with an EN AW-7075 aluminium alloy with the chemical composition shown in Table 1 [28]. The ingots were cast in semi-industrial conditions and then homogenised.

### 2.2. Methods

The research presented in this paper was carried out in several stages. In the first stage, plastometric tests of the analysed aluminium alloy were carried out. In the second stage, the results of the plastometric tests were approximated using the Hensel–Spittel equation [29,30,31], determining its coefficients. The study analysed three mathematical material models of the tested alloy developed with varying degrees of accuracy. The aim of this stage of the research was to determine the impact of the accuracy of the material models used on the most important parameters of the extrusion process: extrusion force and metal flow, with an impact on the geometric stability of the product.

In the next stage of research, the developed mathematical models were then implemented into the material database of the QForm-Extrusion^®^ programme: QForm UK 10.2.1 [32], which was applied for theoretical analysis of the industrial extrusion process.

In the final stage of the work, industrial verification of the extrusion process was performed by using a hydraulic press with a maximum force of 28 MN and a container diameter of 7 inches. The main parameters analysed were the extrusion force, the temperature of the extruded metal, the direction of the metal flow, and the geometric stability of the extruded tubes. The tubes obtained were optically scanned in 3D using a The scanner GOM Atos Core 200 (Lenso, Poznań, Poland).

#### 2.2.1. Plastometric Testing–GLEEBLE 3800 Metallurgical Process Simulator–Uniaxial Compression Test

**Figure 1:**
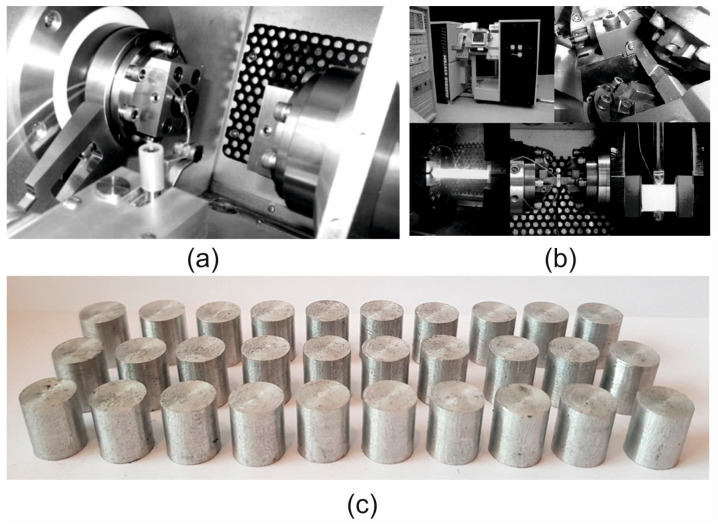
Test station for determining the rheological properties of aluminium alloys: (**a**) test chamber of the Hydrawedge module of the GLEEBLE 3800 simulator; (**b**) Gleeble 3800 physical simulator; (**c**) cylindrical aluminium samples with a diameter of 10 mm and a height of 12 mm.

Plastometric tests of the 7075 aluminium alloy, based on which its rheological properties were determined, and the stress–strain function coefficients were selected, were carried out in uniaxial compression tests using a Gleeble 3800 simulator (Dynamic Systems Inc., Poestenkill, NY, USA), (Figure 1a,b) [33]. The compression tests were performed using a special measuring unit installed in the Hydrawedge system [34,35]. The special design of the test head and the use of two independent hydraulic systems made it possible to omit the acceleration and deceleration phases of the shaping tool during the deformation of the specimens. These technical solutions made it possible to reduce the temperature difference along the length of the sample to below 3 °C [34]. In addition, the Hydrawedge system allowed for very accurate execution of the programmed experiment [33]. The anvil, which was used to apply the deformation, reached the set deformation speed in a very short time and maintained it until the end of the deformation, maintaining the precisely set value of partial and total deformations, even at very high deformation speeds [34,35]. I was also shown in the work of Dyja [36]. 

The following relationships were used to determine the actual strain ε, strain rate $\dot{\varepsilon },$ and yield stress $\sigma_{p}$ during compression [34]:

(1) $$\varepsilon =\left|ln\frac{h_{1}}{h_{0}}\right|$$

(2) $$\dot{\varepsilon }=ln\frac{\varepsilon }{∆t}$$

(3) $$\sigma_{p}=\frac{4\cdot F\cdot h}{h_{0}\cdot \pi \cdot d_{0}^{2}}$$

where $h_{0}$, and, ${\;h}_{1}$ is the initial and final height of the sample, ∆t is the deformation time, F is the force value measured during the test, h is the instantaneous height of the sample, and d_0_ is the initial diameter of the sample.

Plastometric tests were planned in such a way as to enable the development of a yield stress function and its coefficients for deformation conditions characteristic of the extrusion process. Plastometric tests were conducted for the following parameters:

- Temperature: 450 °C, 480 °C, 510 °C;
- Deformation rate: 0.05 s^−1^, 0.5 s^−1^, 1 s^−1^;
- Actual deformation: max. 1.2.

The tests were conducted in a vacuum at a constant temperature of the deformed sample. Cylindrical samples with a diameter of d = 10 mm and a height of h = 12 mm were used for the tests (Figure 1c). In order to minimise friction between the front surfaces of the samples and the surfaces of the tools, graphite washers and a special graphite-based lubricant were used. Two K-type thermocouples (NiCr-NiAl), welded to the side surface of the sample, were used to record and control temperature changes. The samples were heated at a constant rate of 5 °C/s to the set temperature, held at this temperature for 10 s, and then deformed.

#### 2.2.2. Approximation of Plastometric Test Results–Development of Dedicated Material Models

In available computer programmes designed to solve problems related to the plastic flow of metal or to calculate force using the finite element method, the yield stress σ_p_ values depend on the assumed yield stress function (model). Most often, the yield stress is described by the relationship σ_p_ = (ε,($\dot{\varepsilon }$,T)). Many mathematical functions (models) are used to mathematically describe changes in σ_p_ as a function of deformation, temperature, and deformation rate, which can be found in sources such as [15,16,29,30,31,34,35,36,37,38,39,40,41,42,43,44,45,46,47]. In [29], 17 plastic strain functions (models) were analysed in order to find the relationship that most accurately reflects the actual metal flow curves for hot working conditions. Selected plastic strain functions (models) most commonly used to approximate plastometric test results are presented in Table 2.

In this paper, the Hensel–Spittel equation (function no. 20 (Table 2), which can be transformed into the form of Equation (21). This relationship is often used to determine the value of σ_p_ in computer programmes for numerical modelling of plastic working processes [17]. This relationship has also been repeatedly used by the authors in plastometric studies and numerical modelling of real technological processes, giving good results [20,46,47].

(21) $$\sigma_{p}=A\cdot e^{m_{1}\cdot T}\cdot T^{m_{9}}\cdot \varepsilon^{m_{2}}\cdot e^{\frac{m_{4}}{\varepsilon }}\cdot {\left(1+\varepsilon \right)}^{m_{5}\cdot T}\cdot e^{m_{7}\cdot \varepsilon }\cdot {\dot{\varepsilon }}^{m_{3}}\cdot {\dot{\varepsilon }}^{m_{8}\cdot T}$$

where $\sigma_{p}$—yield stress, *T*—temperature, ε—real strain, $\dot{\varepsilon }$—strain rate, *A*, *m*_1_–*m*_9_—coefficients.

As for the selection of three different material models, they were chosen to represent different weights of constants in the Hensel-Spittel equation for the parameters of temperature (T), deformation (ε) and deformation rate ($\dot{\varepsilon }$) in the context of their impact on the yield stress level. In particular, the material models differed primarily in constants A, m_3_ and m_7_ (Table 3). Thus, constant A in the Hensel-Spittel equation at the deformation temperature is smallest for material model no. 2. The constants m_3_ and m_7_ in the Hensel-Spittel equation, located at the deformation rate and deformation, respectively, are highest for material model no. 1. In the case of the least accurate material model no. 3, we had the largest constant A in the Hensel-Spittel equation at the deformation temperature, which means that the extrusion temperature had the greatest influence of those analysed on the level of yield stress formed during hot forming in the process of extruding difficult-to-form aluminium alloy 7075.

#### 2.2.3. FEM Numerical Modelling of Extrusion Process

The QForm-Extrusion programme and three prepared mathematical material models were applied to analyse the extrusion process of 7075 aluminium alloy tubes of Ø50 × 3 mm with the use of a porthole die. A combined Lagrangian–Eulerian approach was used to describe the hot, deformed material’s motion. This approach combines two basic formulations, the Eulerian and Lagrangian approaches. The Eulerian approach adapts a stationary mesh that allows the accuracy of metal flow prediction to be significantly improved and reduces simulation time. The Lagrangian approach makes it possible dynamically movable mesh to animate the profile flow after the bearing exit. These features, consolidated by coupled thermal and mechanical tasks, allow the precise distribution of the profile velocities and extrusion force to be obtained.

**Figure 2:**
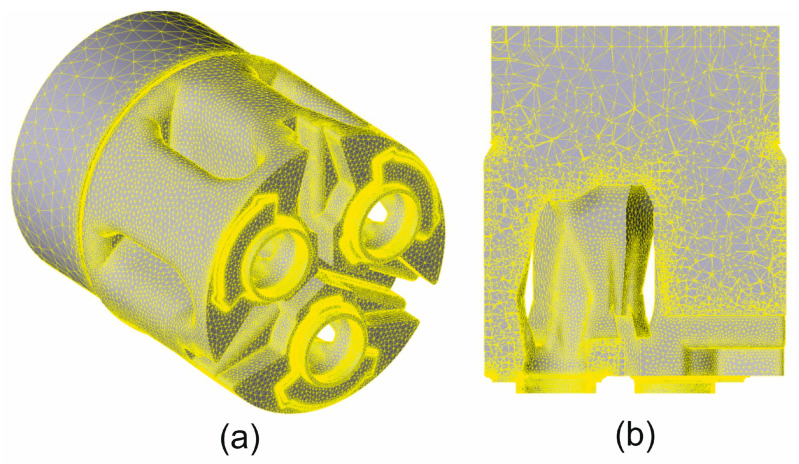
The geometrical model of the extruded material with mesh elements: (**a**) side view showing the billet and the extruded material filling the die gaps; (**b**) cross-sectional view showing the extruded material in the die inlet channels.

The first step in the FEM numerical modelling process was the preparation of properly designed 3D models of the billet and tools with the use of the CAD Solid Works programme. To obtain high-quality results, the mesh element sizes were locally varied. High-density mesh element factors with appropriate element size factors were applied for areas with high plastic deformation (Figure 2).

**Figure 3:**
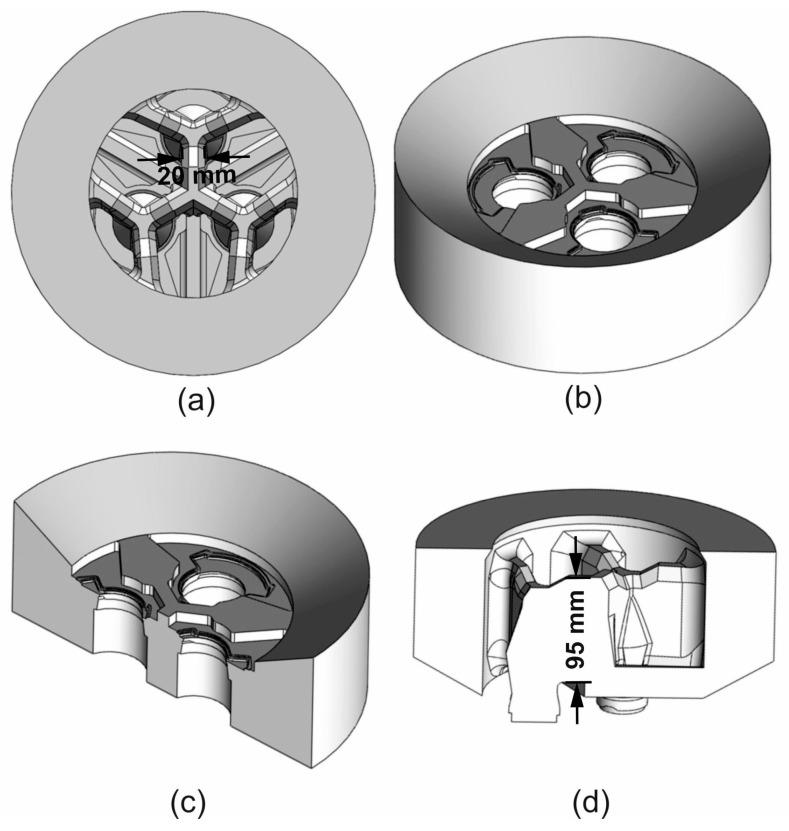
Three-dimensional CAD model of the designed porthole die for extrusion of tubes of Ø50 × 3 mm from 7075 alloy in the cross-sectional view (**a**) and in the bottom view (**b**), in the cross-section (**c**) and in the cross-section with the bridge dimensions marked (height and thickness) (**d**).

The geometry of the porthole die was designed to minimise deformation resistance in the extrusion process and thus obtain a reduction in extrusion force, the elastic deflection of the die, an improvement in metal flow, and dimensional accuracy of the profile (Figure 3). To this end, the following design improvements were introduced:

- Three-arm thick bridges with a thickness of 20 mm and relatively long pins with a length of 95 mm.
- Maximum opening of the die inlet channels—connection of the pins to the body only by means of the bridge arms.
- Use of a feeder with an inlet diameter of Ø178 mm (7″) distributing metal to inlet channels with a diameter of Ø190 mm in order to reduce force and maximise the supply to the outer areas of the profile (difficult flow due to long and thick bridges and distance from the centre).
- Improvement of the mandrel design—reinforcement of the body in the area of connection with the bridges and undercutting/profiling in the area of the welding chambers to facilitate metal flow there.
- Dedicated three-stage pre-chamber increasing the speed of metal flow to the die clearance under the bridges and in the outer area.

The second step of the FEM modelling was the preparation and implementation of the material models for the alloy 7075, which, after approximation, was expressed by the constitutive Hensel–Spittel equation (Equation (1)). The base was the result of the plastometric compression tests conducted using the Gleeble 3800 simulator, which allowed the stress–true strain equation to be obtained as a function of temperature and strain rate. The paper analyses three mathematical material models of the alloy under study, developed with varying degrees of accuracy. The aim of this stage of research was to determine the impact of the accuracy of the material models used on the most important parameters of the extrusion process—extrusion force and temperature of the extruded material—as well as the uniformity of metal flow from the die opening in relation to the dimensional deviations of the extruded Ø50 × 3 mm tubes from the 7075 aluminium alloy.

The third step of the FEM modelling was defining the boundary conditions. The friction model used allowed us to take into account the adhesion effect between aluminium and steel. In the simulations performed on the contact surface of the deformed metal and the tool, the friction model defined by Levanov [43] was adopted.

(22) $$F_{t}=m\cdot \frac{\sigma_{p}}{\sqrt{3}}\cdot \left(1-e^{\left(-1.25\cdot \frac{\sigma_{n}}{\sigma_{p}}\right)}\right)$$

where *m*—friction factor, $\sigma_{n}$– normal contact pressure,$\;\sigma_{p}$—yield stress.

The equation is a combination of the constant friction model and the Coulomb friction model. The second term in parentheses in Equation (1) takes into account the effect of normal pressure on the surface contact. For high-pressure values, the expression approximates the conditions specified by the constant-friction model. For low pressure values, on the other hand, it defines a linear approximation dependent on the normal stress at the surface contact. All the technological parameters of the FEM calculated extrusion process of tubes of Ø50 × 3 mm from 7075 alloy are presented in Table 4.

Two temperatures: 480 °C and 510 °C were proposed based on plastometric tests on a Gleeble physical simulator, which showed that temperatures of 450 °C and 480 °C are safe in terms of the loss of cohesion of the hot-formed 7075 aluminium alloy, while 510 °C is the critical temperature, the exceeding of which results in the loss of cohesion of the hot-formed 7075 aluminium alloy. A temperature representative of a safe hot plastic-forming process was adopted for the tests: 480 °C—the maximum permissible temperature due to the minimization of the plastic forming force for the difficult-to-deform 7075 alloy. The critical temperature was also adopted: 510 °C—the minimum limit temperature, the exceeding of which results in cracks in the hot-formed material.

#### 2.2.4. Industrial Verification

**Figure 4:**
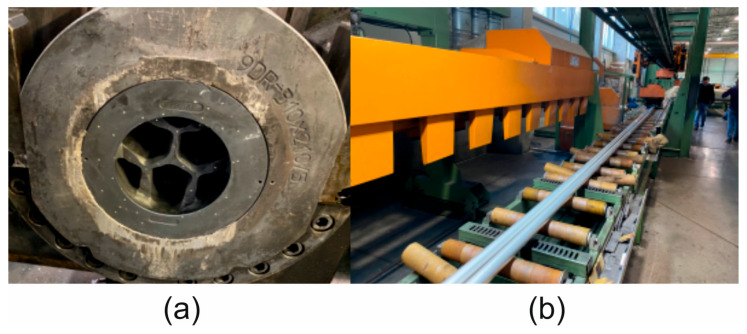
Industrial trials of extrusion of Ø50 × 3 mm tubes made of 7075 aluminium alloy: (**a**) designed porthole die; (**b**) hydraulic press with a 7-inch container diameter and 28 MN pressure force.

Under industrial conditions, tests were carried out on a hydraulic press with a container diameter of 7 inches and a pressure force of 28 MN to extrude Ø50 × 3 mm tubes made of 7075 aluminium alloy using a specially designed porthole die (Figure 4a,b).

**Figure 5:**
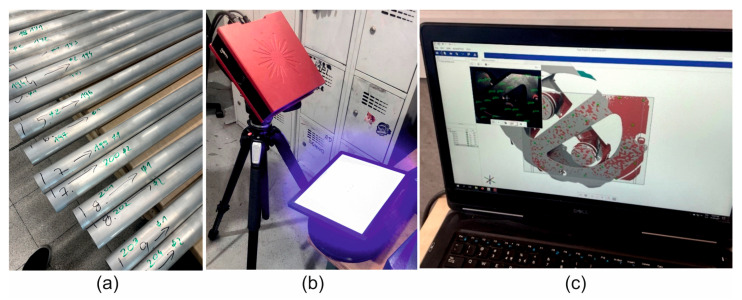
Station for 3D optical scanning of extruded tubes using a GOM ATOS scanner—(**a**) samples of extruded tubes Ø50 × 3 mm made of 7075 aluminium alloy; (**b**) GOM ATOS scanner; (**c**) software for visualisation and processing of measurement data.

Extrusion tests were conducted for two different ingot heating temperatures: 480 °C and 510 °C, and for a fixed ram speed corresponding to the metal flow rate from the die opening at approximately 1.5 m/min. The basic technological parameters of the extrusion process, such as the extrusion force and temperature of the extruded tube as a function of the stem stroke, were recorded using strain gauges, optical pyrometers, and a measurement data acquisition system. A comparison was made of the geometry of the initial section of the profile, the so-called ‘nose piece’, which allowed the characteristics and manner of metal flow from the die opening to be determined. For the purposes of this work, an assessment was made of the manner of metal flow, including the unevenness associated with the arrangement of the openings and the die design. The tubes obtained for a billet heating temperature of 480 °C were subjected to 3D optical scanning using a GOM ATOS scanner (Figure 5) to determine the dimensional deviations in wall thickness and the dimensional deviations in outer and inner diameter.

## 3. Results

### 3.1. Analysis of Plastometric Test Results and Development of Material Model Coefficients for Aluminium 7075

**Figure 6:**
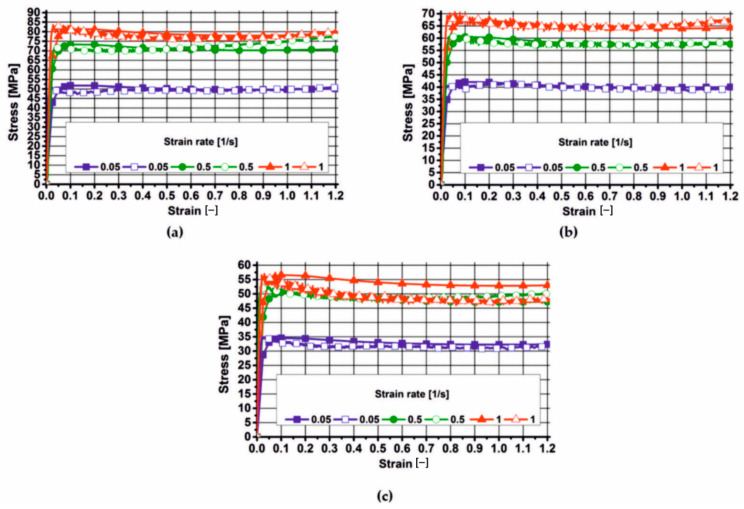
Actual and approximated plastic flow curves of 7075 aluminium using material model no. 1: (**a**) temperature 450 °C; (**b**) temperature 480 °C; (**c**) temperature 510 °C.

**Figure 7:**
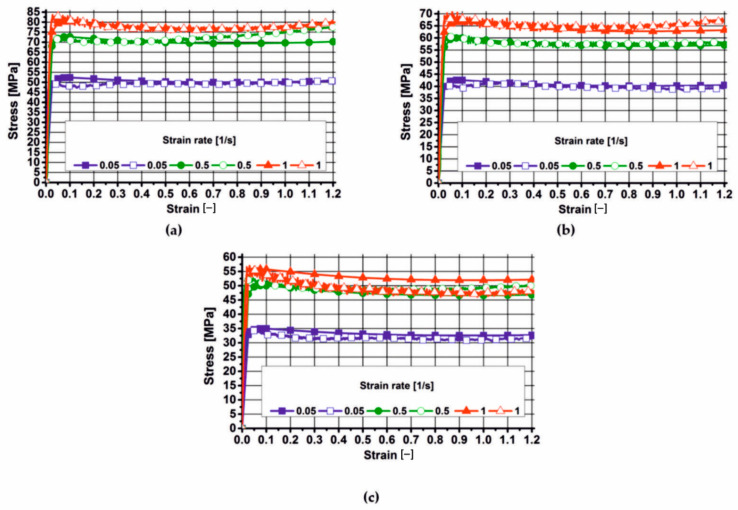
Actual and approximated plastic flow curves of 7075 aluminium using material model no. 2: (**a**) temperature 450 °C; (**b**) temperature 480 °C; (**c**) temperature 510 °C.

**Figure 8:**
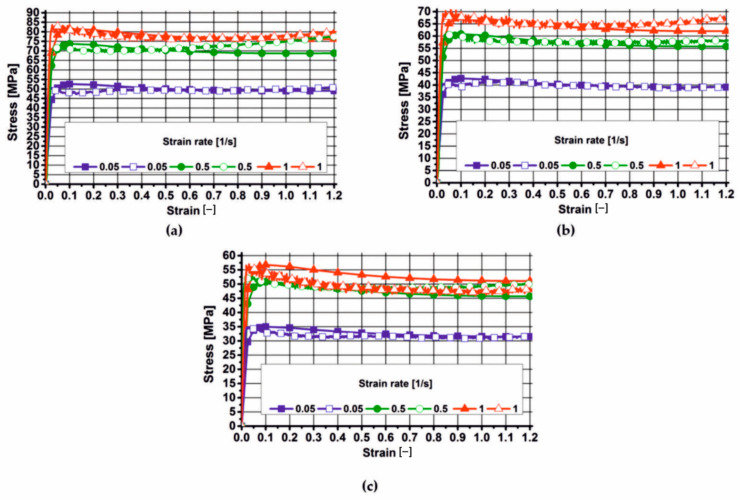
Actual and approximated plastic flow curves of 7075 aluminium using material model no. 3: (**a**) temperature 450 °C; (**b**) temperature 480 °C; (**c**) temperature 510 °C.

This chapter presents the results of the plastometric tests of aluminium 7075, which were approximated with varying degrees of accuracy using the Hansel–Spittel Equation (21). The actual and approximated stress–strain curves for aluminium 7075, in the tested temperature and strain rate range, are presented in Figure 6, Figure 7 and Figure 8. These figures also show the coefficients of the corresponding material models for the tested alloy.

After approximating the results of plastometric tests (with varying degrees of accuracy), the coefficients of Equation (21) were determined. The values of these coefficients are presented in Table 5, Table 6 and Table 7.

By analysing the actual plastic flow curves of alloy 7075 (empty symbols) at a temperature of 450 °C, it was found that during deformation at a deformation rate of 0.05 s^−1^, the yield stress slightly decreased after reaching its maximum value and then (after exceeding a deformation of 0.1) remained constant until the end of the deformation process. During the deformation of the tested material at a deformation rate of 0.5 s^−1^, the actual values of yield stress (empty symbols) after reaching a maximum and a slight decrease, and after exceeding a deformation of 0.3, showed a continuous increase with an increase in the value of the applied deformation. As a result of deforming the 7075 alloy at a rate of 1 s^−1^, the actual values of yield stress also decreased with increasing deformation after reaching their maximum value.

Based on the analysis of the actual plastic flow curves of the tested alloy (open symbols) during deformation at 480 °C, it was found that the material’s microstructure recovered during deformation at a rate of 0.05 s^−1^. The actual yield stress curves of the analysed material (open symbols) during deformation at rates of 0.5 s^−1^ and 1 s^−1^ show a similar pattern. After reaching the maximum value, the yield stress decreased slightly and then slightly increased with increasing applied strain. When the analysed alloy was deformed at a strain rate of 5 s^−1^, recrystallization occurred in its microstructure.

Based on the analysis of the actual plastic flow curves (open symbols) of the 7075 alloy deformed at 510 °C, it can be concluded that, regardless of the applied strain rate, recrystallization of the microstructure occurred in the material.

Based on the analysis of the plastometric test results, it was found that during the deformation of alloy 7075 at 450 °C, increasing the strain rate from 0.05 s^−1^ to 0.5 s^−1^ caused an increase in the actual yield stress values (on average) by approximately 45%. Increasing the strain rate from 0.5 s^−1^ to 1 s^−1^ caused an increase in the actual yield stress values (on average) by approximately 8%.

During deformation of alloy 7075 at 480 °C, increasing the strain rate from 0.05 s^−1^ to 0.5 s^−1^ increased the actual yield stress values (on average) by about 45%. In turn, increasing the strain rate from 0.5 s^−1^ to 1 s^−1^ increased the actual yield stress values (on average) by about 12%.

Analysing the actual plastic flow curves of the 7075 alloy deformed at 510 °C, it was found that increasing the strain rate from 0.05 s^−1^ to 0.5 s^−1^ resulted in an increase in the yield stress (on average) by approximately 58%. Increasing the strain rate from 0.5 s^−1^ to 1 s^−1^ did not significantly affect the yield stress level of the 7075 alloy.

Analysing the effect of temperature on the yield stress level of the tested aluminium alloy, it was found that it decreased with the increase in temperature (for the appropriate strain rate) at which the tested material was deformed, and that the value of yield stress increased with the increase in strain rate (for the appropriate temperature).

### 3.2. Results of Numerical Modelling of the Extrusion Process

**Figure 9:**
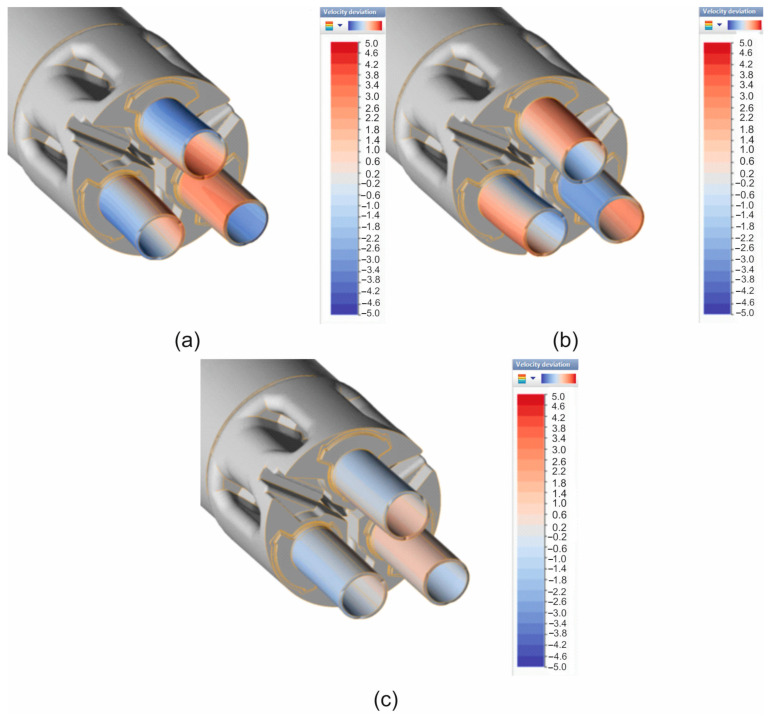
Distribution of velocity deviation while extruding tubes of dimensions of Ø50 × 3 mm made from 7075 aluminium alloy through porthole die for billet temperature of 480 °C and three different assumed material models—(**a**) material model no. 1, (**b**) material model no. 2, (**c**) material model no. 3.

Figure 9 shows the numerically calculated FEM distribution of the variation in metal flow velocity during the extrusion of 7075 aluminium alloy tubes with dimensions of Ø50 × 3 mm through a 3-opening porthole die for a billet heating temperature of 480 °C and three different material models. In the case of material model no. 1 (Figure 9a), the metal tends to flow slightly faster in the inner areas of the extruded tubes (it is estimated that the metal flows approx. 3–3.8% faster than the average speed), which results in a slight bending of the products towards the outside. This may indicate that the metal supply from the 3-stage pre-chambers located in the outer parts of the die is too weak, resulting in faster metal flow in the central part of the die. In the case of material model no. 2 (Figure 9b), we have the exact opposite situation, i.e., the metal tends to flow slightly faster in the outer areas of the extruded tubes (it is estimated that the metal flows approx. 3–3.4% faster than the average speed), which results in a slight inward bending of the products.

However, when using material model no. 3 (Figure 9c) in FEM numerical calculations, we see the most even metal flow from the die opening, with a slightly faster flow in the central areas of the extruded tubes (it is estimated that the metal flows approx. 0.6–1% faster than the average speed), resulting in an almost straight, geometrically stable product. This type of metal flow may indicate a correctly designed porthole die, in particular, in the design of the bridge and inlet channels, as well as the pre-chambers and calibration belts of the die.

**Figure 10:**
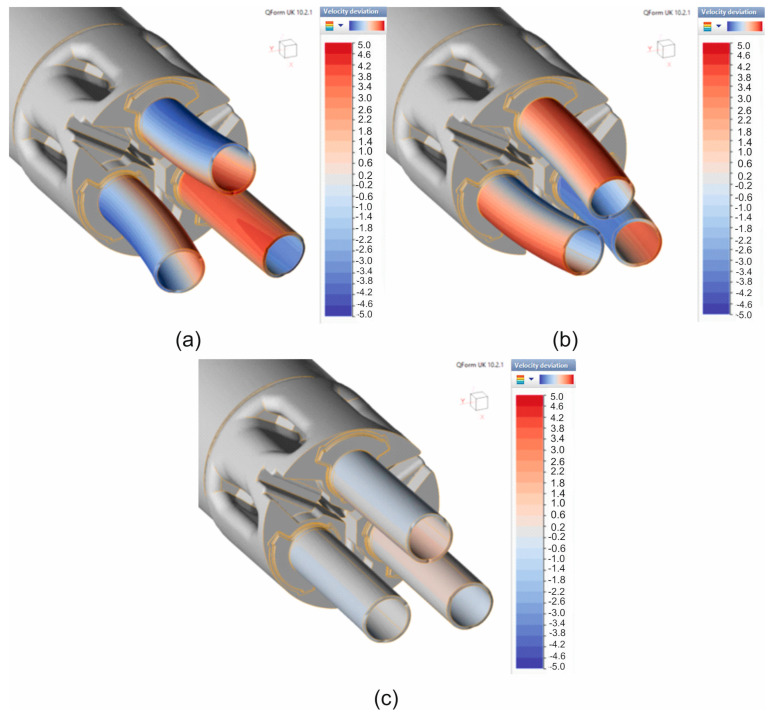
Distribution of velocity deviation while extruding tubes of dimensions of Ø50 × 3 mm made from 7075 aluminium alloy through porthole die for billet temperature of 510 °C and three different assumed material models—(**a**) material model no. 1, (**b**) material model no. 2, (**c**) material model no. 3.

In the case of a higher heating temperature of 510 °C, a similar trend can be observed, but with a more varied metal flow when using material models no. 1 and no. 2. It is estimated that the metal flow rate may differ from the average value by up to approx. 5%. As a result of this flow pattern, the extruded tubes bend strongly outwards when material model no. 1 is used (Figure 10a) or inwards when material model no. 2 is used (Figure 10b). When using material model no. 3, there is almost no variation in the metal flow velocity in the die clearance (it is estimated that the flow in the central areas of the extruded tubes is minimally faster by approx. 0.2–0.6% compared to the average value), resulting in a straight, geometrically stable product (Figure 10c).

**Figure 11:**
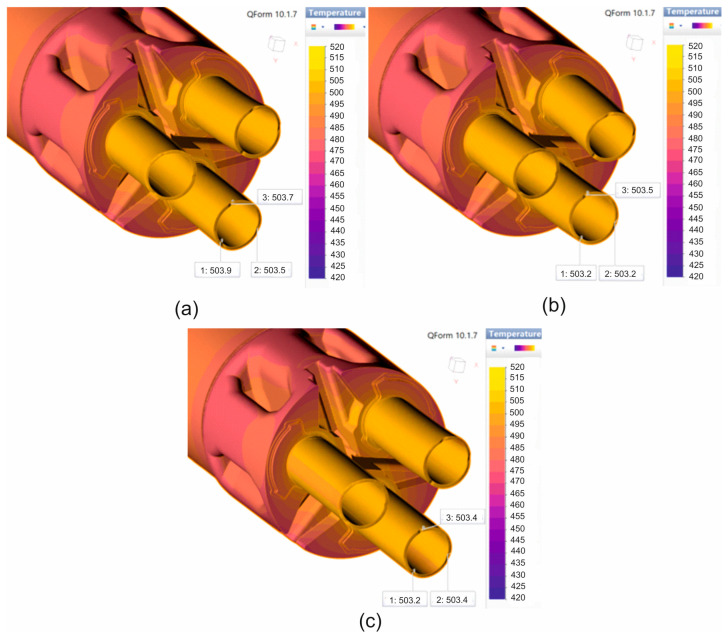
Distribution of material temperature while extruding tubes of dimensions of Ø50 × 3 mm made from 7075 aluminium alloy through porthole die for billet temperature of 480 °C and three different assumed material models—(**a**) material model no. 1, (**b**) material model no. 2, (**c**) material model no. 3.

**Figure 12:**
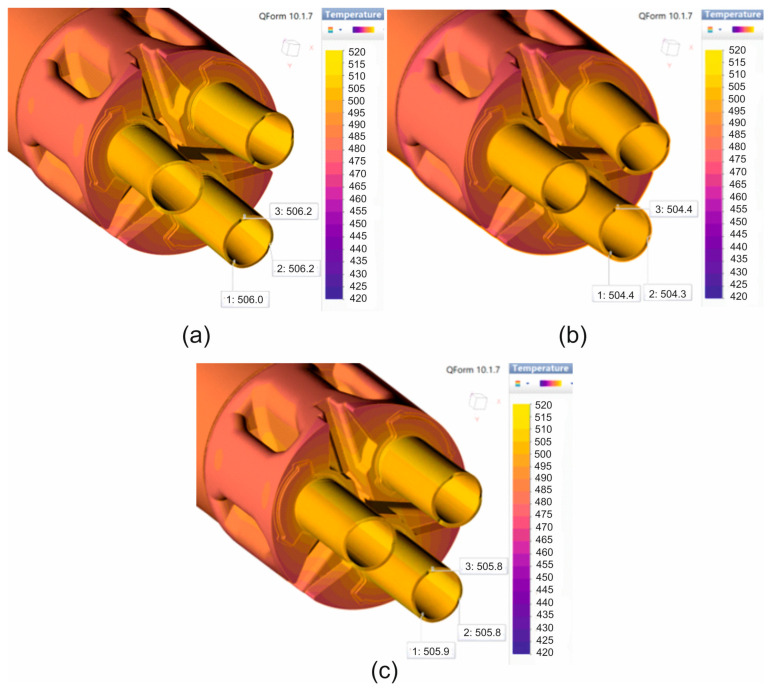
Distribution of material temperature while extruding tubes of dimensions of Ø50 × 3 mm made from 7075 aluminium alloy through porthole die for billet temperature of 510 °C and three different assumed material models—(**a**) material model no. 1, (**b**) material model no. 2, (**c**) material model no. 3.

Figure 11 and Figure 12 show the numerically calculated FEM temperature distributions of the material during the extrusion of 7075 aluminium alloy tubes with dimensions of Ø50 × 3 mm through a 3-hole porthole die, for the billet heating temperatures of 480 °C and 510 °C, and for three different material models. In general, for a billet heating temperature of 480 °C, very similar values of the extruded material temperature were obtained after leaving the die hole, regardless of the material model used, at around 503–504 °C (Figure 11). However, in the case of a higher ingot heating temperature of 510 °C, the temperatures of the extruded tubes after leaving the die opening were several degrees Celsius higher, at around 506 °C for material models no. 1 and no. 3 (Figure 12a,c) and approx. 504 °C for material model no. 2 (Figure 12b). In summary, it can be concluded that for both sprue heating temperatures, the temperature of the extruded tubes does not exceed the critical value of 510 °C determined in tests on the Gleeble simulator. However, it should be taken into account that the obtained temperature distributions refer to the initial phase of the extrusion process, and the temperature of the product may still increase slightly as the process progresses.

**Figure 13:**
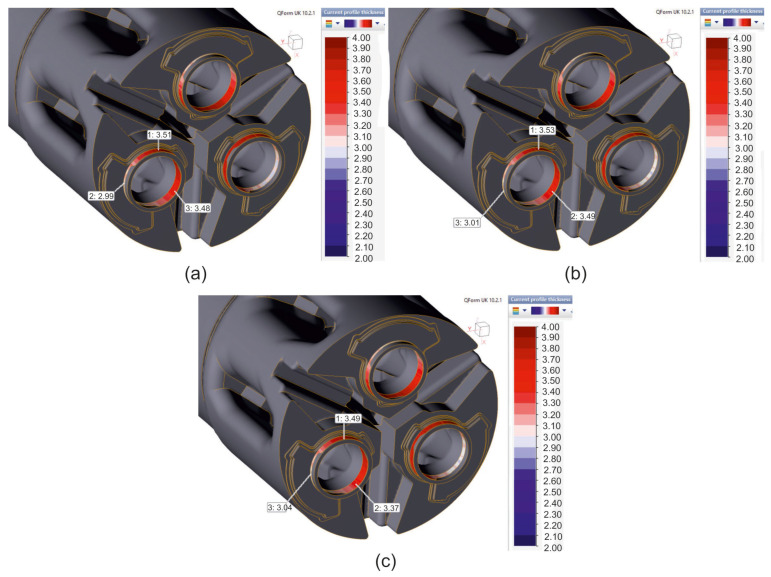
FEM calculated predicted product’s wall thicknesses while extruding tubes of dimensions of Ø50 × 3 mm made from 7075 aluminium alloy through a porthole die for a billet temperature of 480 °C and three different assumed material models—(**a**) material model no. 1, (**b**) material model no. 2, (**c**) material model no. 3.

FEM calculated predicted product’s wall thicknesses while extruding tubes of dimensions of Ø50 × 3 mm from 7075 aluminium alloy through porthole die for the billet temperature of 480 °C and three different assumed material models were shown in Figure 13. Generally, regardless of the material model used, a relatively large variation in the wall thickness of the extruded tube around its circumference is observed, ranging from 2.99 to 3.53 mm. In the inner parts of the tubes, i.e., those located closer to the centre of the die, a significant thickening of the extruded tubes was found—the positive dimensional deviation of the wall thickness ranges from 0.37 to 0.53 mm (the largest positive deviation for material models no. 1: 0.51 mm and no. 2: 0.53 mm), which exceeds the permissible value specified by the European standard. In turn, in the inner parts of the tubes, i.e., those located outside the die, it was found that the tubes have a wall thickness close to the nominal value of 3.0 mm—from the minimum wall thinning: wall thickness 2.99 mm (material model no. 1; Figure 13a) up to the minimum wall thickening to 3.01 mm (material model no. 2; Figure 13b) and 3.04 mm (material model no. 3; Figure 13c). Such a distribution of the wall thickness of extruded tubes may indicate significant axial elastic deflection of the die during the extrusion process and, as a consequence, an increase in the die clearance in the central part of the die (internal areas of the tubes).

**Figure 14:**
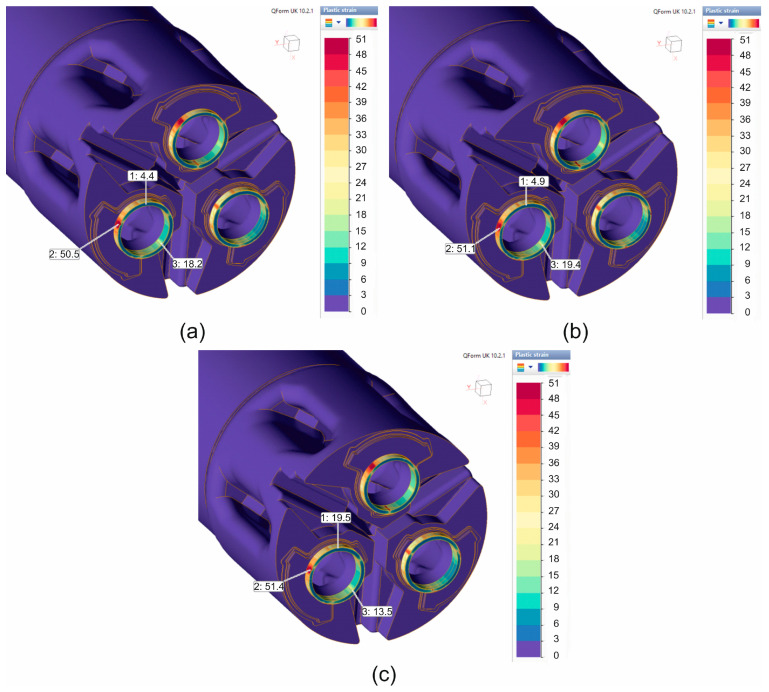
FEM calculated plastic strain while extruding tubes of dimensions of Ø50 × 3 mm made from 7075 aluminium alloy through a porthole die for a billet temperature of 480 °C and three different assumed material models—(**a**) material model no. 1, (**b**) material model no. 2, (**c**) material model no. 3.

This is confirmed by the plastic deformation distributions on the circumference of the extruded tubes (Figure 14), where a fairly significant variation in plastic deformation values on the circumference of the tubes can be observed—strongly localised large deformations in the area of pre-chambers. The greatest variation in plastic deformation around the circumference of extruded tubes was found, similarly to the variation in tube wall thickness, for material models no. 1 in the range of 4.4 to 50.5 (Figure 14a) and no. 2 in the range of 4.9 to 51.1 (Figure 14b), while it was slightly smaller for material model no. 3, in the range of 13.5 to 51.4 (Figure 14c).

**Figure 15:**
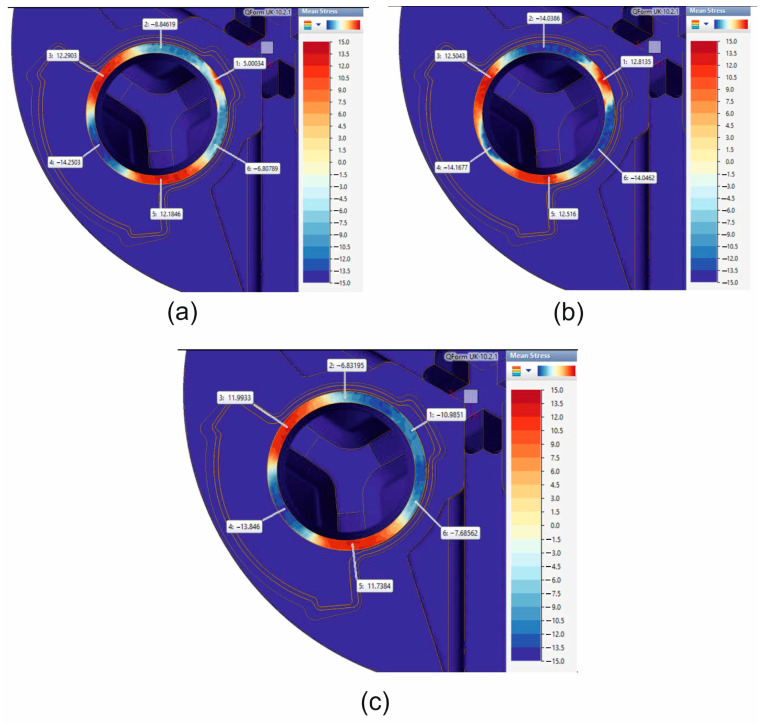
FEM calculated mean stress while extruding tubes of dimensions of Ø50 × 3 mm made from 7075 aluminium alloy through porthole die for billet temperature of 510 °C and three different assumed material models—(**a**) material model no. 1, (**b**) material model no. 2, (**c**) material model no. 3.

FEM was used to calculate mean stress distributions while extruding tubes of dimensions of Ø50 × 3 mm from 7075 aluminium alloy through a porthole die for a billet temperature of 510 °C, and three different assumed material models are shown in Figure 15.

The least favourable stress state, with three positive tensile stresses (12.51 MPa, 12.50, and 12.81 MPa) that are responsible for material failure, occurs when material model no. 2 is used in FEM numerical calculations. In the case of material models no. 3, there is one negative compressive stress and two positive tensile stresses, which are more conducive to plastic deformation without loss of material cohesion (Figure 15).

**Figure 16:**
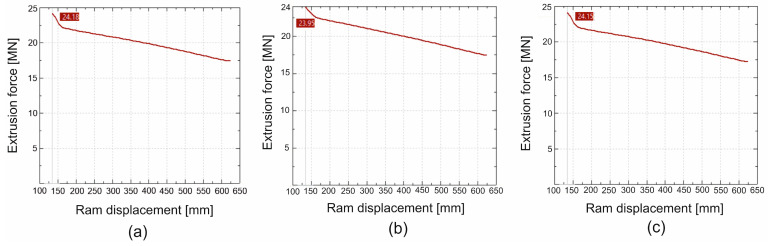
FEM calculated extrusion force vs. ram displacement while extruding tubes of dimensions of Ø50 × 3 mm made from 7075 aluminium alloy through porthole die for billet temperature of 480 °C and three different assumed material models—(**a**) material model no. 1, (**b**) material model no. 2, (**c**) material model no. 3.

**Figure 17:**
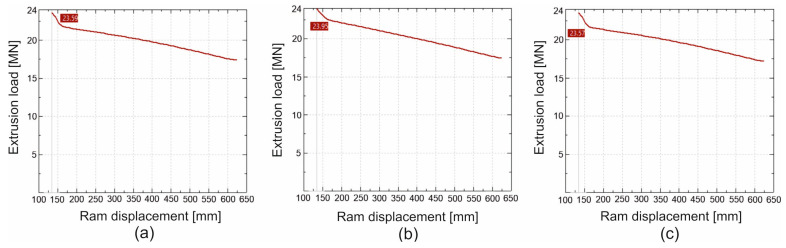
FEM calculated extrusion force vs. ram displacement while extruding tubes of dimensions of Ø50 × 3 mm made from 7075 aluminium alloy through porthole die for billet temperature of 510 °C and three different assumed material models—(**a**) material model no. 1, (**b**) material model no. 2, (**c**) material model no. 3.

In Figure 16 and Figure 17 we can see the FEM-calculated extrusion force vs. ram displacement while extruding tubes of dimensions of Ø50 × 3 mm from 7075 aluminium alloy through a porthole die for billet temperatures of 480 °C and 510 °C and three different assumed material models—(a) material model no. 1, (b) material model no. 2, (c) material model no. 3. We can observe the force characteristics typical for the extrusion process, with maximum force at the beginning of the process and decreasing force as the press ram moves forward, reaching its minimum at the end of the process.

### 3.3. Results of Industrial Verification

**Figure 18:**
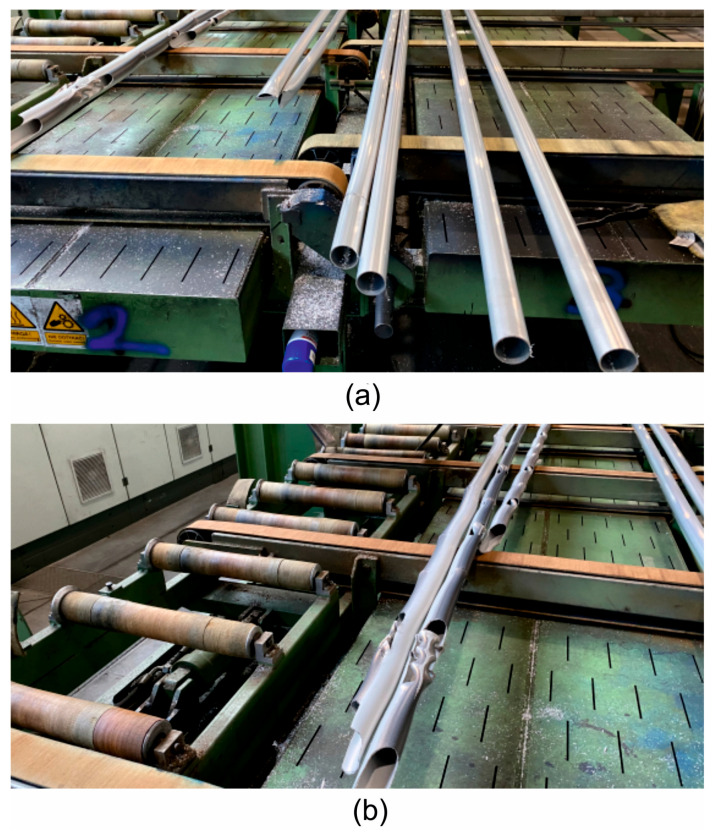
Photographic documentation of extrusion tests of Ø50 × 3 mm tubes made of 7075 aluminium alloy—(**a**) tubes after extrusion at a billet temperature of 480 °C, (**b**) tubes after extrusion at a billet temperature of 510 °C.

In the case of extruded tubes for a billet heating temperature of 480 °C, a product with adequate surface quality was obtained (Figure 18a), while in the case of extruded tubes for a billet heating temperature of 510 °C, there was a loss of material cohesion and numerous surface cracks and tears in the extruded tubes (Figure 18b). Loss of material cohesion during extrusion indicates that the material’s limit temperature has been exceeded and/or that unfavourable tensile stresses have occurred in the die clearance, which reduce the permissible speed of metal flow from the die opening.

**Figure 19:**
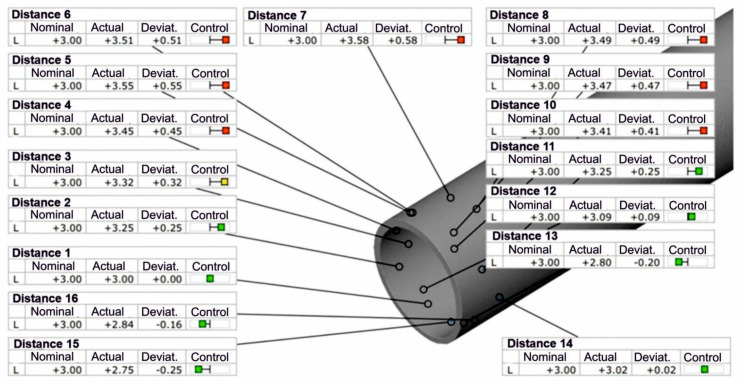
The real wall thickness deviations for tubes of dimensions of Ø50 × 3 mm made from 7075 aluminium alloy extruded through porthole die (for billet temperature of 480 °C) (Red indicates dimensional deviations that are unacceptable according to the standard).

The results of 3D optical scanning of the extruded Ø50 × 3 mm tubes made of 7075 aluminium alloy (for a sprue heating temperature of 480 °C) using porthole dies indicated a high geometric instability of the product. In the case of tube wall thickness, we encountered very large, unacceptable dimensional deviations resulting from the large variation in the speed of metal flow from the die opening, as well as from the potentially large elastic deflection of the die. Figure 19 shows the wall thickness deviations around the circumference of the extruded tube, with predominantly positive deviations, indicating, above all, a significant thickening of the walls of the extruded tubes. The largest positive deviation was found to be +0.58 mm and the largest negative deviation was −0.25 mm. The European standard PN-EN 755-8: 2016 allows for a variation in wall thickness (tube eccentricity) of up to ±7%, while the actual variation in the wall thickness of extruded tubes in the analysed case is 13.83%, which is almost twice the permissible value. In the case of deviations in the external and internal diameter of extruded tubes, the standard allows for deviations of ±0.35 mm, while the actual deviations exceed even 2 mm, both positive and negative (Figure 19).

The simplest solution seems to be to increase the height of the bridges, although care must be taken not to cause excessive friction resistance and ultimately increase the force in the extrusion process and reduce the permissible speed of metal flow from the die opening. The authors of this article plan to perform further FEM numerical calculations using a modified porthole die design, together with experimental verification under industrial conditions.

**Figure 20:**
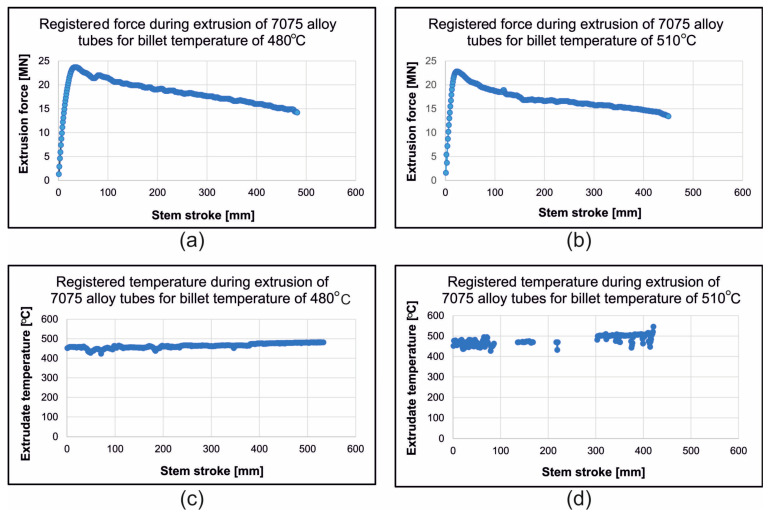
The registered extrusion technological parameters while extruding tubes of dimensions of Ø50 × 3 mm made from 7075 aluminium alloy through porthole die for different billet temperatures—(**a**) extrusion force for billet temperature of 480 °C, (**b**) extrusion force for billet temperature of 510 °C, (**c**) extruded material temperature for billet temperature of 480 °C, (**d**) extruded material temperature for billet temperature of 510 °C.

In Figure 20, the registered extrusion technological parameters while extruding tubes with dimensions of Ø50 × 3 mm, made from 7075 aluminium alloy, through a porthole die at different billet temperatures are presented. In the case of the extrusion variant for a lower ingot heating temperature of 480 °C, the maximum and minimum extrusion forces were recorded at 23.7 MN and 14.2 MN, respectively, and the temperature of the extruded material was in the range of 460–495 °C. In the case of the extrusion variant for a higher ingot heating temperature of 510 °C, the maximum and minimum extrusion forces were recorded at 22.8 MN and 13.4 MN, respectively, and the temperature of the extruded material was in the range of 480–525 °C. Due to numerous ruptures of extruded tubes at a sprue temperature of 510 °C, the pyrometric measurement of the temperature of the extruded material was not continuous but intermittent.

## 4. Discussion

In Table 8, the individual process/product parameters that were compared in various tests are shown, including those for the plastometric tests on Gleeble, the numerical FEM calculations, and the experimental verification in the industrial tube extrusion process.

**Figure 21:**
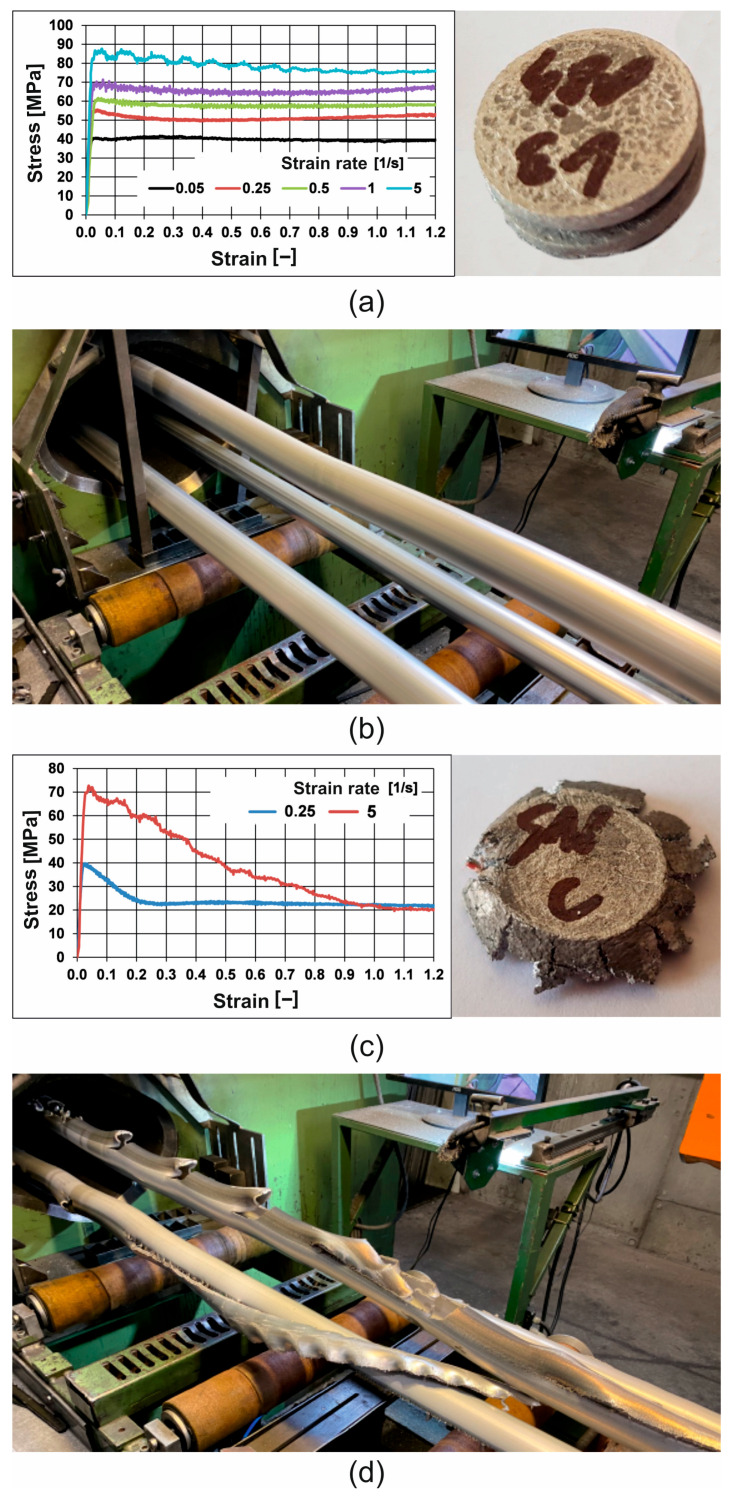
A comparison of the results of the plastometric tests on the 7075 alloy in the Gleeble simulator and the results of the industrial process of the extrusion of tubes of dimensions Ø50 × 3 mm made from the 7075 alloy by using porthole dies: (**a**) stress–strain curves determined on the Gleeble device at 480 °C with different strain rates, and the sample after compression at 480 °C with a strain rate of 5 s^−1^; (**b**) image of extruded tubes of dimensions of 50 × 3 mm made from alloy 7075 for a billet heating temperature of 480 °C; (**c**) stress–strain curves determined on the Gleeble device at 510 °C with different strain rates, and the sample after compression at 510 °C with a strain rate of 5 s^−1^ (**d**) image of extruded tubes of dimensions of 50 × 3 mm made from alloy 7075 for a billet heating temperature of 510 °C.

Preliminary tests showed that the tested 7075 aluminium alloy lost its plastic deformation capacity at 540 °C. The loss of material cohesion manifested itself in a sharp drop in yield stress starting in the initial stage of the deformation process. As can be seen from the data presented in Figure 21c, during deformation at a temperature of 510 °C, the material showed some instability, i.e., it retained its cohesion during deformation at low deformation rates of 0.05 s^−1^, but during deformation at a high deformation rate of 5 s^−1^, it lost its cohesion at the beginning of the deformation process. It should be noted here that industrial extrusion processes of 7075 aluminium alloys take place at high deformation rates in the range of 100–101 s^−1^, so at such deformation speeds, at an extrusion temperature of 510 °C, a loss of material cohesion and a sharp drop in plastic flow stress can be expected. This is confirmed by the image of the extruded tubes in question for a billet heating temperature of 510 °C, where numerous surface cracks and transverse and longitudinal tears in the tubes can be observed, resulting precisely from the loss of material cohesion after exceeding the limit temperature (Figure 21d). At the same time, the extruded tubes in question for a billet heating temperature of 480 °C retain material cohesion and good surface quality even at high deformation speeds (Figure 21b). This comparison of plastometric test results from the Gleeble simulator for different temperatures and deformation speeds (Figure 21a,c) and test results from the industrial extrusion process of the Ø50 × 3 mm tubes in question on porthole dies for different billet heating temperatures of 480 °C and 510 °C (Figure 21b,d) indicate that the Gleeble simulator tests correctly predict the behaviour of the material in the actual technological process (material deformability) with the determination of the plastic forming limit temperature. This is of great practical importance, as it enables the correct selection of technological parameters for the industrial plastic-forming process and avoids time-consuming and costly implementation trials.

**Figure 22:**
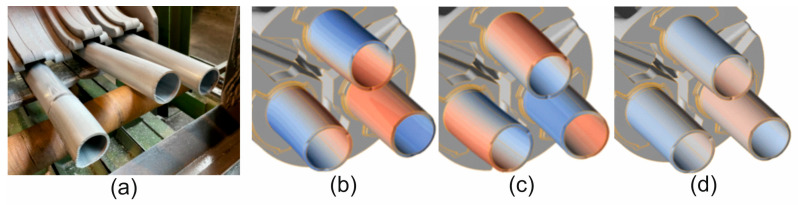
Comparison of metal flow in the industrial extrusion process of Ø50 × 3 mm tubes of 7075 aluminium alloy using porthole dies for a billet heating temperature of 480 °C: (**a**) actual bending of the ends of extruded tubes and (**b**–**d**) numerically calculated FEM bending of the ends of extruded tubes for 3 different mathematical material models (Red indicates faster metal flow from the die hole, while blue indicates slower flow).

**Figure 23:**
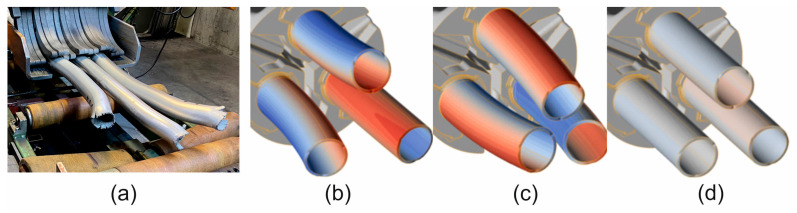
Comparison of metal flow in the industrial extrusion process of Ø50 × 3 mm tubes of 7075 aluminium alloy using porthole dies for a billet heating temperature of 510 °C: (**a**) actual bending of the ends of extruded tubes and (**b**–**d**) numerically calculated FEM bending of the ends of extruded tubes for 3 different mathematical material models (Red indicates faster metal flow from the die hole, while blue indicates slower flow).

A comparison of metal flow in the industrial extrusion process of Ø50 × 3 mm tubes of 7075 aluminium alloy using porthole dies for billet heating temperatures of 480 °C and 510 °C was presented in Figure 22 and Figure 23. As can be seen, the greatest similarity in the manner in which metal flows out of the die opening and, consequently, in the geometric stability of the extruded tubes occurs for material model no. 1 for both analysed ingot heating temperatures. In particular, a high degree of consistency can be observed between the FEM calculation results for material model no. 1 and the actual extrusion process for a higher billet heating temperature of 510 °C, where a fairly strong outward bending of the extruded tubes is observed, indicating a faster metal flow in the central segment of the die. In stark contrast to material model no. 1, material model no. 2 indicates a completely different flow of metal from the die opening, resulting in a strong inward bend of the extruded tubes. In the case of material model no. 3, for a billet heating temperature of 510 °C, we obtain uniform flow and a geometrically simple product, which is not reflected in reality. For a lower heating temperature of 480 °C, a very even flow of metal from the die holes and a geometrically stable product with slight bending of the extruded tubes towards the outside were observed during industrial tests. In principle, for all three material models analysed, a high degree of consistency between the results of the FEM numerical calculations and the extrusion tests was found, with material model no. 1 being the most favourable.

**Figure 24:**
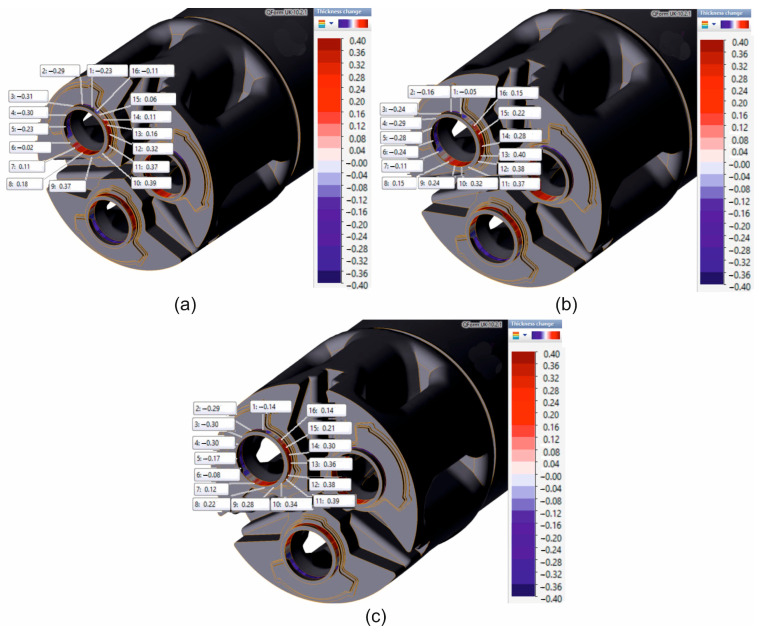
FEM numerically calculated dimensional deviations in the wall thickness of Ø50 × 3 mm tubes extruded from 7075 aluminium alloy using porthole dies for a billet heating temperature of 480 °C and 3 different mathematical material models: (**a**) material model no. 1; (**b**) material model no. 2; and (**c**) material model no. 3.

**Figure 25:**
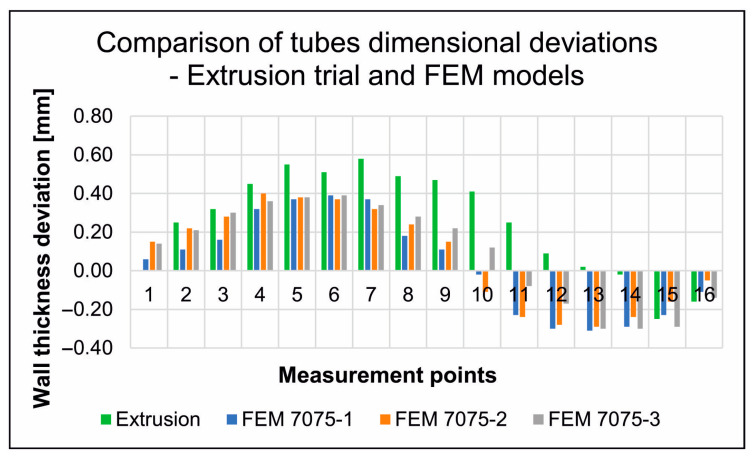
Comparison of wall thickness deviations for tubes Ø50 × 3 mm extruded from 7075 aluminium alloy using porthole dies for a billet heating temperature of 480 °C: industrial extrusion process and FEM numerical calculations (3 different mathematical material models).

FEM numerically calculated dimensional deviations in the wall thickness of Ø50 × 3 mm tubes extruded from 7075 aluminium alloy using porthole dies for a billet heating temperature of 480 °C, and the results from the three different mathematical material models are shown in Figure 24. For each of the analysed material models, numerical FEM calculations of the hot extrusion process revealed significant variations in the wall thickness of the 7075 aluminium alloy tubes, with a high positive dimensional deviation of +0.41 mm and a high negative dimensional deviation of −0.31 mm. However, positive wall thickness deviations clearly predominate on the circumference of the extruded tubes. In Figure 25, the comparison of wall thickness deviations was presented (industrial extrusion process and FEM numerical calculations) for tubes Ø50 × 3 mm extruded from 7075 aluminium alloy for a billet heating temperature of 480 °C. In general, a high degree of convergence was found in the distribution of wall thickness deviations around the circumference of the tubes in question for the results of numerical FEM calculations, regardless of the material model used, and for the data obtained from 3D optical scanning of tubes extruded under industrial conditions. However, in terms of the maximum wall thickness deviations obtained in reality (positive deviation +0.58 mm and negative deviation −0.25 mm), the closest values are the FEM results for material model no. 2 (positive deviation +0.40 mm and negative deviation −0.29 mm). On the other hand, the very nature of the transition of dimensional deviations in the wall thickness of extruded tubes from positive to negative is most closely reflected by material model no. 3.

**Figure 26:**
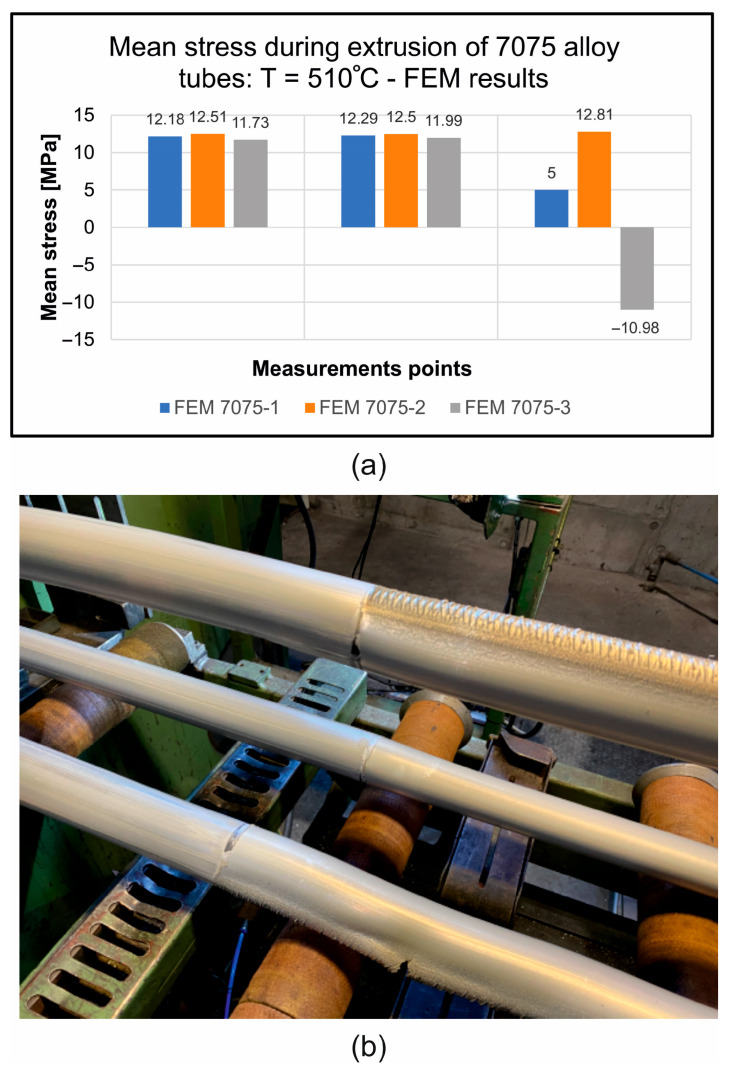
A summary of numerically calculated FEM stress conditions in the die clearance, three different tube segments during the extrusion of 50 × 3 mm tubes made of 7075 aluminium alloy for three different material models (**a**) with images of the surfaces of the tubes extruded under industrial conditions (**b**).

Figure 26 presents a summary of numerically calculated FEM stress conditions in the die clearance (in three different tube segments) during the extrusion of 50 × 3 mm tubes made of 7075 aluminium alloy for three different material models (Figure 26a) with images of the surfaces of the tubes extruded under industrial conditions (Figure 26b).

In the first segment of the extruded tubes, only tensile stresses were obtained, which are responsible for the material’s cohesion loss. In the second segment of the extruded tubes, all positive tensile stresses were again recorded, creating a risk of a loss of cohesion in the plastically formed material. Finally, in the third segment of the extruded tubes, unfavourable positive tensile stresses were observed for material models no. 1 and no. 2, with the highest positive stress close to 13 MPa for material model no. 2. and negative compressive stress for the material model no. 3. Figure 1b,c show the surface quality of the extruded tubes in industrial conditions with numerous surface cracks caused by unfavourable tensile stresses. In summary, in the case of material models no. 1 and no. 2, tensile stresses responsible for the loss of material cohesion were found in three out of three segments of the die clearance, which is the closest reflection of actual conditions (for material model no. 3, tensile stresses were obtained in two out of three segments of the die clearance).

**Figure 27:**
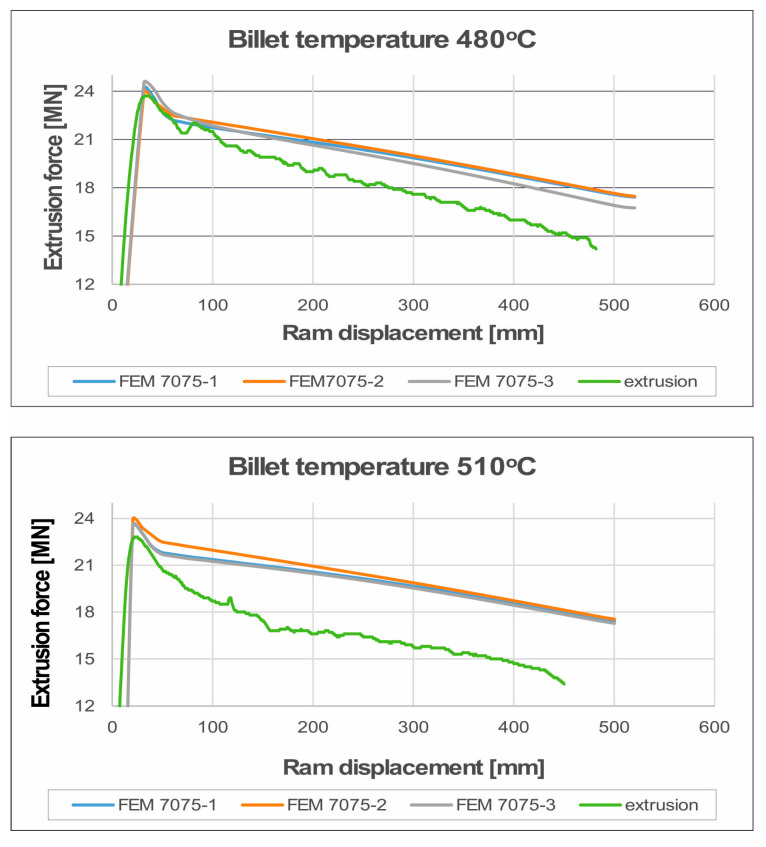
Comparison of the extrusion force as a function of the punch travel—numerically calculated FEM and recorded in industrial tests for extrusion welding of tubes Ø50 × 3 mm made of 7075 aluminium alloy for ingot heating temperatures of 480 °C (**upper** figure) and 510 °C (**lower** figure).

Comparison of the extrusion force as a function of the punch travel–numerically calculated FEM as recorded in industrial tests during the extrusion welding of tubes of Ø50 × 3 mm made of 7075 aluminium alloy for different ingot heating temperatures is presented in Figure 27. In general, the maximum extrusion force values from industrial trials are very similar to those obtained in the FEM calculations for the three different material models used. For a billet heating temperature of 480 °C, the maximum extrusion force obtained from FEM numerical calculations that was closest to the corresponding maximum value from the real extrusion process was obtained from material model no. 2 (23.95 MN in FEM and 23.70 MN in real extrusion process: Figure 27 upper). Similarly, with regard to the minimum extrusion force obtained as a result of FEM numerical calculations, the closest value to the corresponding minimum value from the real extrusion process refers to material model no. 2 (16.70 MN in FEM, while 14.2 MN in the real extrusion process). It is important to note the slight difference in the values of the predicted FEM maximum extrusion force for individual material models, which amounts to only 2.5%. Slightly lower extrusion force values, but also very similar to those from the FEM calculations for the three different material models, were obtained for a higher billet heating temperature of 510 °C (Figure 27 lower). The closest maximum extrusion force for this increased billet heating temperature was 23.57 MN for FEM and material model no. 3 and 22.80 MN for the real extrusion process. Here, we have an even smaller difference in the predicted FEM maximum extrusion force for individual material models, which was only 1.6%.

In summary, it should be noted that the numerical FEM calculations of the maximum extrusion force of the aluminium alloy 7075 tubes in question are very accurate: 96.5–99.0% for a billet heating temperature of 480 °C, and 95.0–96.6% for a billet heating temperature of 510 °C. The prediction of the minimum extrusion force at the end of the extrusion process was less accurate, but this force has no practical significance because, unlike the maximum extrusion force, it does not determine the possibility of performing the extrusion process on a press with given force capabilities. The numerically calculated FEM minimum extrusion force was approximately 18–20% higher than that in the actual extrusion process. It can be concluded that the analysed material models were unable to effectively model the significant softening of the material during extrusion at the minimum force, which was significantly lower in the actual extrusion process.

In general, for all three analysed material models, a high degree of convergence was found between the results of the FEM numerical calculations and the extrusion trials, with material model no. 1 proving the most favourable. This is primarily due to the small differences between the actual yield stress values and the values approximated using material model no. 1. This is crucial because yield stress (and its distribution within the material volume) determines the material flow pattern, particularly the deformation of the blank during die clearance, which in turn directly affects the direction of metal bending as it exits the die opening. Furthermore, characterising the metal flow pattern over a wide temperature range allows us to determine the mechanisms of elastic deformation, the values of mean and effective stress, and friction on the die surface. These values, varying for individual alloys, determine the behaviour of the die during the process, primarily by deflecting the bridges and mandrel bodies, and especially the bearings, and by decalibration of the die clearance itself, which directly translates into the geometric accuracy of the product.

**Figure 28:**
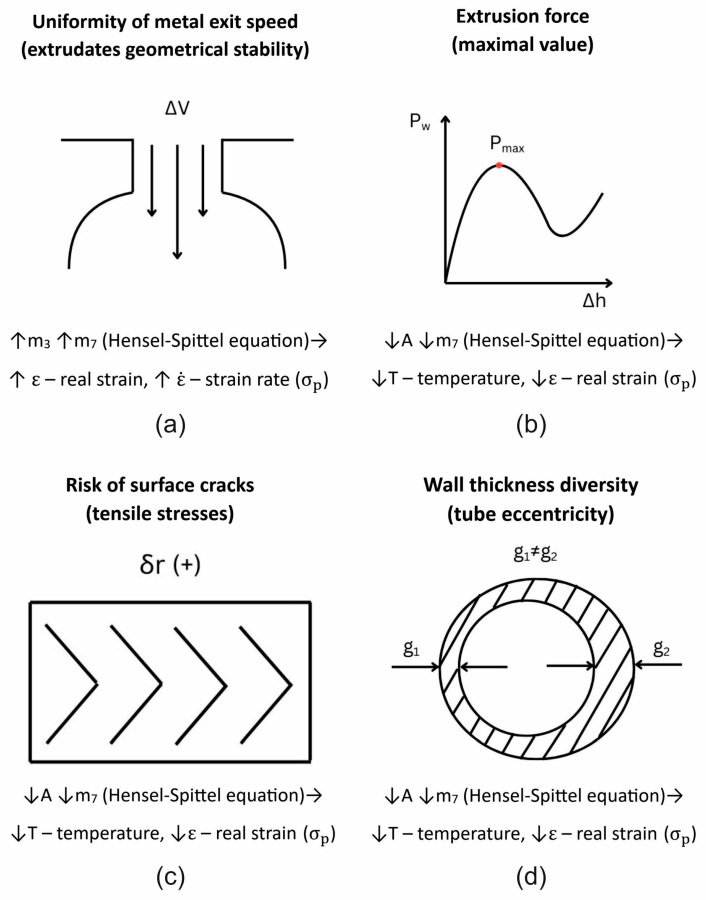
The mind map showing the accuracy of reflecting the actual state of process and product parameters using FEM numerical calculations: (**a**) material model no. 1, (**b**–**d**) material model no. 2.

The mind map (Figure 28) shows the accuracy of reflecting the actual state of process and product parameters using FEM numerical calculations with the assignment of appropriate coefficients from the Hensel–Spittel equation for individual material models and their impact on plastic strain.

Thus, the constants m_3_ and m_7_ in the Hensel–Spittel equation, representing the deformation rate and deformation, respectively, are highest for material model no. 1. Material model no. 1 allows for the most accurate prediction of the uniformity of metal flow in extruded tubes, which would mean the relatively strongest influence of the real strain and strain rate on the yield stress. The constant A in the Hensel–Spittel equation at the deformation temperature is smallest for material model no. 2. Material model no. 2 allows for the most accurate prediction of the extrusion force, wall thickness instability, and extrudate cracking, which would mean a relatively weak influence of the extrusion temperature and real strain on the yield stress. In the case of the least accurate material model, no. 3, we had the largest constant A in the Hensel–Spittel equation at the deformation temperature, which means that the extrusion temperature had the greatest influence out of all those analysed on the level of yield stress of the hot-extruded difficult-to-form aluminium alloy 7075.

## 5. Conclusions

1. All three analysed mathematical material models of 7075 aluminium alloy intended for hot extrusion ensured high accuracy of FEM numerical calculations in terms of maximum extrusion force and dimensional deviations of the wall thickness of a Ø50 × 3 mm tube. The accuracy of the maximum extrusion force calculations, which is the most important factor from the point of view of the feasibility of the extrusion process on a hydraulic press with a given pressure force, was 98.1% for material model no. 1, 99.0% for material model no. 2, and 96.5% for material model no. 3 (for a billet temperature of 480 °C), and 96.5% for material model no. 1, 95% for material model no. 2, and 96.6% for material model no. 3 (for a billet temperature of 510 °C).
2. In the case of wall thickness deviations of the extruded tube, all three material models allowed for the correct prediction of high positive deviations ranging from 0.32 mm to 0.40 mm (actual maximum positive dimensional deviation 0.55 mm) and moderate negative deviations ranging from −0.29 mm to −0.31 mm (actual maximum negative dimensional deviation −0.25 mm). However, the closest was material model no. 2.
3. When predicting the uniformity of metal flow during extrusion and the resulting geometric stability of the extruded tube, the only material model for 7075 aluminium alloy that has been positively verified under industrial conditions is material model no. 1. Only for this material model was it possible to correctly predict the level of variation in the speed of metal flow from the die opening and the manner of bending of the extruded tubes after leaving the die for different billet heating temperatures.
4. The most adequate material model for aluminium alloy 7075 in terms of correctly predicting positive tensile stresses in the die clearance responsible for surface cracks in the extruded product is model no. 2 (for a limit temperature of 510 °C, it allowed for the prediction of unfavourable positive tensile stresses in three areas of the die clearance).
5. The rheological properties of aluminium alloy 7075 intended for extrusion through porthole dies, determined on a Gleeble physical simulator, allowed for accurate prediction of the conditions of material cohesion loss during extrusion (surface cracking of hot-extruded Ø50 × 3 mm tubes) under industrial conditions. For the tubes in question, made of 7075 aluminium alloy, the limit temperature of the extruded material was determined to be 510 °C, above which the surface quality of the extruded product was lost.

## Figures and Tables

**Table 1:** Chemical composition of investigated EN AW-7075 aluminium alloy [28].

Designation	Melt Analysis, Mass %								
	Si	Fe	Cu	Mn	Mg	Cr	Zn	Ti	Zr
EN AW-7075	0.10	0.21	1.54	0.00	2.39	0.20	5.89	0.02	0.15

**Table 2:** Selected functions (models) of yield stress used to approximate the results of plastometric tests [29].

Function (Model) Form	
$\sigma_{p}=\alpha_{1}\cdot \varepsilon^{\alpha_{2}}$	(4)
$\sigma_{p}=\alpha_{1}+\alpha_{2}\cdot \varepsilon$	(5)
$\sigma_{p}=\alpha_{1}+\alpha_{2}\cdot \varepsilon +\alpha_{3}\cdot \varepsilon^{2}$	(6)
$\sigma_{p}=\alpha_{1}+\alpha_{2}\cdot \varepsilon +\alpha_{3}\cdot \varepsilon^{2}+\alpha_{4}\cdot \varepsilon^{3}$	(7)
$\sigma_{p}=\alpha_{1}+\alpha_{2}\cdot \varepsilon +\alpha_{3}\cdot \varepsilon^{2}+\alpha_{4}\cdot \varepsilon^{3}+\alpha_{5}\cdot \varepsilon^{4}$	(8)
$\sigma_{p}=\alpha_{1}+\alpha_{2}\cdot \varepsilon +\alpha_{3}\cdot \varepsilon^{2}+\alpha_{4}\cdot \varepsilon^{3}+\alpha_{5}\cdot \varepsilon^{4}+\alpha_{6}\cdot \varepsilon^{5}$	(9)
$\sigma_{p}=\alpha_{1}+\alpha_{2}\cdot \varepsilon^{\alpha_{3}}$	(10)
$\sigma_{p}=\alpha_{1}+\alpha_{2}\cdot \varepsilon +\alpha_{3}\cdot \dot{\varepsilon }+\alpha_{4}\cdot T$	(11)
$\sigma_{p}=\alpha_{1}\cdot \varepsilon^{\alpha_{2}}{\dot{\varepsilon }}^{\alpha_{3}}\cdot e^{-T\cdot \alpha_{4}}$	(12)
$\sigma_{p}=\alpha_{1}+\alpha_{2}\cdot \varepsilon +\alpha_{3}\cdot \dot{\varepsilon }+\alpha_{4}\cdot T+\alpha_{5}\cdot \varepsilon \cdot \dot{\varepsilon }+\alpha_{6}\cdot \varepsilon \cdot T+\alpha_{7}\cdot T\cdot \dot{\varepsilon }+\alpha_{8}\cdot \varepsilon^{2}+\alpha_{9}\cdot {\dot{\varepsilon }}^{2}+\alpha_{10}\cdot T^{2}$	(13)
$\sigma_{p}=\alpha_{1}+\alpha_{2}\cdot \varepsilon +\alpha_{3}\cdot \dot{\varepsilon }+\alpha_{4}\cdot T+\alpha_{5}\cdot \varepsilon^{2}+\alpha_{6}\cdot {\dot{\varepsilon }}^{2}+\alpha_{7}\cdot T^{2}$	(14)
$\sigma_{p}=\alpha_{1}+\alpha_{2}\cdot \varepsilon +\alpha_{3}\cdot \dot{\varepsilon }+\alpha_{4}\cdot T+\alpha_{5}\cdot \varepsilon \cdot \dot{\varepsilon }\cdot T^{2}$	(15)
$\sigma_{p}=\alpha_{1}\cdot \varepsilon^{\alpha_{2}}\cdot e^{\alpha_{3}\cdot \varepsilon }\cdot {\dot{\varepsilon }}^{\alpha_{4}}\cdot e^{\alpha_{5}\cdot T}$	(16)
$\sigma_{p}=\alpha_{1}+\alpha_{2}\cdot \varepsilon +\alpha_{3}\cdot \dot{\varepsilon }+\alpha_{4}\cdot T+\alpha_{5}\cdot \varepsilon^{2}+\alpha_{6}\cdot {\dot{\varepsilon }}^{2}+\alpha_{7}\cdot T+\alpha_{8}\cdot \varepsilon^{3}$	(17)
$\sigma_{p}=\alpha_{1}+\alpha_{2}\cdot \varepsilon +\alpha_{3}\cdot \dot{\varepsilon }+\alpha_{4}\cdot T+\alpha_{5}\cdot \varepsilon^{2}+\alpha_{6}\cdot {\dot{\varepsilon }}^{2}+\alpha_{7}\cdot T+\alpha_{8}\cdot \varepsilon^{3}+\alpha_{9}\cdot \varepsilon^{4}$	(18)
$\sigma_{p}=\alpha_{1}\cdot \varepsilon^{\alpha_{2}+\alpha_{3}\cdot T+\alpha_{4}\cdot \dot{\varepsilon }}\cdot e^{\alpha_{5}\cdot \varepsilon }\cdot {\dot{\varepsilon }}^{\alpha_{6}+\alpha_{7}\cdot T}\cdot e^{\alpha_{8}\cdot T}$	(19)
$\sigma_{p}=\alpha_{1}\cdot \varepsilon^{\alpha_{2}}\cdot e^{\frac{\alpha_{3}}{\varepsilon }}\cdot e^{\alpha_{4}\cdot \varepsilon }\cdot {\left(1+\varepsilon \right)}^{\alpha_{5}\cdot T}\cdot {\dot{\varepsilon }}^{\alpha_{6}}\cdot {\dot{\varepsilon }}^{\alpha_{7}\cdot T}\cdot T^{\alpha_{8}}\cdot e^{\alpha_{9}\cdot T}$	(20)

where $\sigma_{p}$—yield stress, *T*—temperature, ε—real strain, $\dot{\varepsilon }$—strain rate, $\alpha_{1}$–$\alpha_{10}$—coefficients.

**Table 3:** The assumed different constants A, m_3_ and m_7_ from Hensel-Spittel equation for material models no. 1, 2 and 3.

Coefficients	Material Models		
	No. 1	No. 2	No. 3
A	837	829	841
m_3_	0.046	0.038	0.035
m_7_	0.252	0.242	0.246

**Table 4:** Technological parameters of the extrusion process for Ø50 × 3 mm pipes made of 7075 aluminium alloy used in FEM numerical calculations.

Alloy	7075: 3 material models
Billet dimensions	Ø178 × 800 mm
Billet temperature	480, 500, 510 °C
Container/Die temperature	450 °C
Extrusion ratio	36
Stem velocity	1.0 mm/s
Metal exit speed	1.5 m/min
Friction coefficient	1

**Table 5:** The determined coefficients of Hensel-Spittel Equation – for material model no 1.

A	m_1_	m_2_
837303084.813	−0.0005231499	−0.0609081271
m_3_	m_4_	m_5_
0.04674237654	−0.0093611401	−0.0007896485
m_7_	m_8_	m_9_
0.25286445658	0.00023102114	−2.6106996399
Root Mean Saquare (RMS)		
min.	max.	avg.
1.33	5.30	2.87

**Table 6:** The determined coefficients of Hensel-Spittel Equation – for material model no 2.

A	m_1_	m_2_
829435323.711	−0.00053254322	−0.03745324342
m_3_	m_4_	m_5_
0.03854563213	−0.00414453245	−0.00078813243
m_7_	m_8_	m_9_
0.24257543243	0.00023001211	−2.61043442110
Root Mean Saquare (RMS)		
min.	max.	avg.
1.37	5.95	2.96

**Table 7:** The determined coefficients of Hensel-Spittel Equation – for material model no 3.

A	m_1_	m_2_
841165598.01211	−0.00051006910	−0.05598999450
m_3_	m_4_	m_5_
0.03598785420	−0.00854422150	−0.00089589668
m_7_	m_8_	m_9_
0.24698514778	0.00024589889	−2.61064423880
Root Mean Saquare (RMS)		
min.	max.	avg.
1.16	4.99	2.93

**Table 8:** The individual process/product parameters that were compared in various tests—plastometric tests on Gleeble, numerical FEM calculations, and experimental verification.

Process/Product Parameters	Gleeble	FEM Calculations	Experimental/Extrusion
Critical temperature	+	−	+
Extrusion force	−	+	+
Extrudate wall thickness instability	−	+	+
Uniformity of the metal exit speed	−	+	+
Loss of material cohesion	+	+	+

## Data Availability

The original contributions presented in this study are included in the article. Further inquiries can be directed to the corresponding author.
